# Mitochondrial matrix protein LETMD1 maintains thermogenic capacity of brown adipose tissue in male mice

**DOI:** 10.1038/s41467-023-39106-z

**Published:** 2023-06-23

**Authors:** Anna Park, Kwang-eun Kim, Isaac Park, Sang Heon Lee, Kun-Young Park, Minkyo Jung, Xiaoxu Li, Maroun Bou Sleiman, Su Jeong Lee, Dae-Soo Kim, Jaehoon Kim, Dae-Sik Lim, Eui-Jeon Woo, Eun Woo Lee, Baek Soo Han, Kyoung-Jin Oh, Sang Chul Lee, Johan Auwerx, Ji Young Mun, Hyun-Woo Rhee, Won Kon Kim, Kwang-Hee Bae, Jae Myoung Suh

**Affiliations:** 1 Metabolic Regulation Research Center, Korea Research Institute of Bioscience and Biotechnology (KRIBB), Daejeon, 34141 Republic of Korea; 2 Graduate School of Medical Science and Engineering, KAIST, Daejeon, 34141 Republic of Korea; 3 Department of Chemistry, Seoul National University, Seoul, 08826 Republic of Korea; 4 Neural Circuit Research Group, Korea Brain Research Institute, Daegu, 41068 Republic of Korea; 5 Laboratory of Integrative Systems Physiology, École polytechnique fédérale de Lausanne (EPFL), CH-1015 Lausanne, Switzerland; 6 Department of Functional Genomics, KRIBB School of Bioscience, Korea University of Science and Technology (UST), Daejeon, 34141 Republic of Korea; 7 Digital Biotech Innovation Center, KRIBB, Daejeon, 34141 Republic of Korea; 8 Department of Biological Sciences, KAIST, Daejeon, 34141 Republic of Korea; 9 National Creative Research Center for Cell Plasticity, KAIST Stem Cell Center, Department of Biological Sciences, KAIST, Daejeon, 34141 Republic of Korea; 10 Disease Target Structure Research Center, KRIBB, Daejeon, 34141 Republic of Korea; 11 Biodefense Research Center, KRIBB, Daejeon, 34141 Republic of Korea; 12 School of Medicine, Sungkyunkwan University, Suwon, 16419 Republic of Korea; 13 Department of Developmental and Cell Biology, School of Biological Sciences, University of California, Irvine, CA 92697 USA

**Keywords:** Energy metabolism, Homeostasis, Energy metabolism

## Abstract

Brown adipose tissue (BAT) has abundant mitochondria with the unique capability of generating heat via uncoupled respiration. Mitochondrial uncoupling protein 1 (UCP1) is activated in BAT during cold stress and dissipates mitochondrial proton motive force generated by the electron transport chain to generate heat. However, other mitochondrial factors required for brown adipocyte respiration and thermogenesis under cold stress are largely unknown. Here, we show LETM1 domain-containing protein 1 (LETMD1) is a BAT-enriched and cold-induced protein required for cold-stimulated respiration and thermogenesis of BAT. Proximity labeling studies reveal that LETMD1 is a mitochondrial matrix protein. *Letmd1* knockout male mice display aberrant BAT mitochondria and fail to carry out adaptive thermogenesis under cold stress. *Letmd1* knockout BAT is deficient in oxidative phosphorylation (OXPHOS) complex proteins and has impaired mitochondrial respiration. In addition, BAT-specific *Letmd1* deficient mice exhibit phenotypes identical to those observed in *Letmd1* knockout mice. Collectively, we demonstrate that the BAT-enriched mitochondrial matrix protein LETMD1 plays a tissue-autonomous role that is essential for BAT mitochondrial function and thermogenesis.

## Abstract (web summary)

Brown adipose tissue (BAT) has abundant mitochondria with the unique capability of generating heat via uncoupled respiration. Here, Park et al. identify LETMD1 as a mitochondrial matrix protein enriched in brown adipose tissue (BAT) and reveal a crucial role for it in maintaining brown adipocyte mitochondrial OXPHOS and thermogenesis upon cold stimulus.

## Introduction

Brown adipose tissue (BAT) differs from white adipose tissue (WAT) in that BAT is highly responsive to cold exposure for adaptive thermogenesis. BAT has abundant mitochondria that carry out its unique heat-generating function. Uncoupling protein 1 (UCP1), localized to the mitochondrial inner membrane of brown adipocytes, plays a major role in the thermogenic function of brown adipocytes. When BAT is activated by cold conditions, UCP1 dissipates proton (H^+^) motive force in the form of heat as a transporter of protons generated by the mitochondrial respiratory chain^1^. In this respect, mitochondrial respiration in BAT has an essential role in regulating whole-body energy homeostasis through adaptive thermogenesis in cold stress.

BAT glucose uptake and mitochondrial oxidative activity are increased by cold exposure in rodents^2^ and humans^3^. In activated BAT, a coordinated increase in aerobic energy metabolism and UCP1-mediated uncoupled respiration metabolize nutrients for heat production in non-shivering thermogenesis. BAT transplantation improves glucose tolerance and insulin resistance^4^, and counteracts obesity in mice^5^. Furthermore, in humans, BAT activation increases energy expenditure, reduces body fat mass, and improves whole-body glucose disposal and insulin sensitivity^6,7^. Thus, therapeutic manipulation of BAT activity has emerged as a promising strategy for the treatment of obesity and metabolic disorders^8,9^. To successfully implement this strategy, it is crucial to understand the molecular components that mediate the thermogenic function of BAT mitochondria induced by cold stimulus. However, aside from UCP1, other BAT mitochondrial factors required for uncoupled respiration and thermogenesis under cold stress are largely unknown.

In this study, we surveyed cold-inducible mitochondrial proteins in BAT and showed that LETM1 domain-containing protein 1 (LETMD1) is a BAT-enriched factor localized to the mitochondrial matrix of brown adipocytes. The *Letmd1* gene was initially identified as an oncogene (*HCCR1* or *2*) associated with human cervical cancer and has been found to be highly expressed in various cancer cells, where it is linked to tumor suppressor function^10–14^. Our analysis of *Letmd1* knockout (KO) mice demonstrated that LETMD1 plays an essential role in cold-stimulated respiration and adaptive thermogenesis of BAT. Furthermore, LETMD1-deficient brown adipocytes exhibited dysmorphic mitochondrial ultrastructure and a significant reduction in OXPHOS complex protein levels. Elucidating the role of LETMD1 in BAT thermogenesis contributes to a deeper understanding of molecular mechanisms that enable the functional specialization of mitochondria in thermogenic BAT.

## Results

### Identification of cold-inducible mitochondrial proteins in BAT

To identify candidate genes involved in the thermogenic function of BAT mitochondria, we analyzed publicly available gene expression data for genes that are enriched in BAT, as compared to epididymal WAT (eWAT) (GSE92844)^15^, and induced by cold stimulus (GSE70437)^16^ (Supplementary Fig. 1a). From this analysis, we uncovered a cluster of 144 genes that were both enriched in BAT and induced by cold stimulation (Supplementary Fig. 1a). Among these 144 genes, a subgroup of 31 genes were annotated as mitochondrial genes including *Ucp1* (Supplementary Figs. 1b and 2).

Mitochondrial gene transcript levels and protein levels are frequently discordant, so we performed a complementary analysis with proteomics data^17^. By analyzing the intersection of proteins that are BAT-enriched proteins and also induced by cold, we identified 26 mitochondrial proteins that showed concordant expression at the transcript level (Supplementary Figs. 1c and 2). After combining the results of the transcriptomic and proteomic analyses, three candidate genes, *Ucp1*, *Letmd1*, and *Acsl5* were identified (Supplementary Fig. 1d). UCP1 is a well-established mitochondrial protein required for BAT thermogenesis and ACSL5 has also been reported as an important regulator of whole-body energy metabolism in previous studies^18^. However, in contrast to UCP1 and ACSL5, the role of LETMD1 in BAT has not been explored.

### LETMD1 is a BAT-enriched protein that is induced by cold exposure

**Fig. 1:**
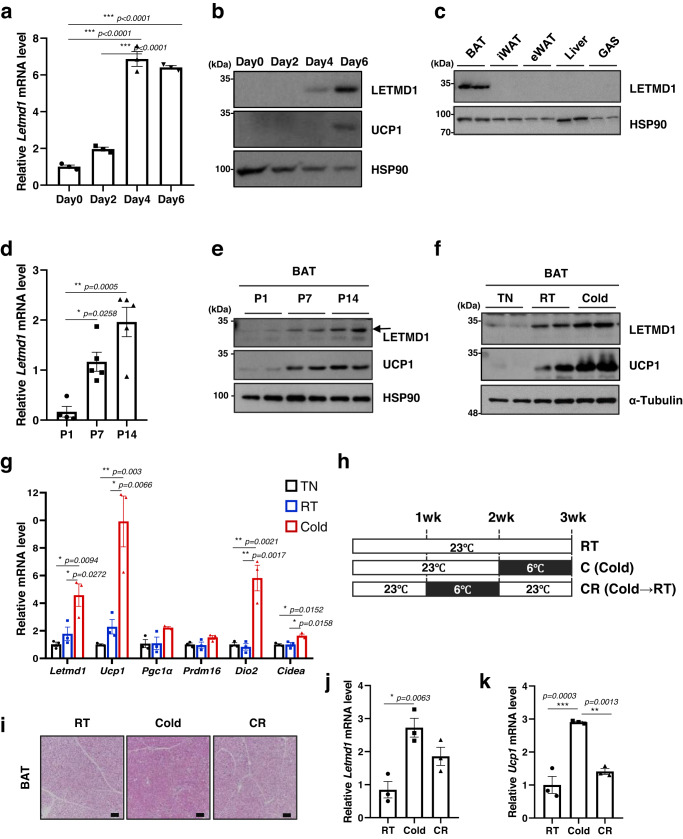
The *Letmd1* gene encodes a brown adipocyte-enriched and cold-inducible protein. **a** mRNA expression of *Letmd1* during brown adipocyte (iBPA) differentiation. Expression is normalized to Rpl32. *n* = 3 per group. **b** Western blots of LETMD1 and UCP1 proteins during brown adipocyte (iBPA) differentiation. HSP90 is a loading control. **c** Western blot of LETMD1 protein in brown adipose tissue (BAT), inguinal white adipose tissue (iWAT), epididymal white adipose tissue (eWAT), liver, and gastrocnemius muscle (GAS) from adult male mice. HSP90 is a loading control. Representative images from three independent repeats. **d** mRNA expression of *Letmd1* from perinatal BAT from postnatal day 1 (P1), day 7 (P7), and day 14 (P14) male mice. n_P1_ = 4, n_P7_ = 5, n_P14_ = 5. Expression is normalized to Rpl32. **e** Western blots of LETMD1 and UCP1 proteins in BAT in the same sets as in (**d**). HSP90 is a loading control. Representative images from three independent repeats. **f** Representative western blots of LETMD1 and UCP1 proteins in BAT from adult male mice after 5 days of exposure to TN (thermoneutral, 30 °C), RT (room temperature, 23 °C), and cold (6 °C). α -Tubulin is a loading control. Representative images from three independent repeats. **g** mRNA expression of *Letmd1* and thermogenic genes in the same sets as in (**f**). Expression is normalized to Rpl32. *n* = 3 per group. **h** Experimental scheme for a cold challenge in adult male mice. **i** H&E staining of BAT sections from mice exposed to cold (6 °C) and mice adapted to room temperature (23 °C) after cold stimulation. Scale bar, 200 μm. Representative images from three independent repeats. mRNA expression of *Letmd1* (**j**) and *Ucp1* (**k**) in the same sets as in (**h**). Expression is normalized to Rpl32. *n* = 3 mice per group. Data presented as mean ± SEM. *p < 0.05, **p < 0.005, ***p < 0.0005. The significance of the results was assessed using one-way ANOVA. Source data are provided as a Source Data file.

To begin to understand the role of LETMD1 in BAT, we examined *Letmd1* gene expression during brown adipocyte differentiation in an immortalized brown preadipocyte (iBPA) cell culture model^19^. During iBPA differentiation into brown adipocytes, *Letmd1* gene expression increased at both RNA and protein levels (Fig. 1a, b). In adult mice, LETMD1 protein was highly enriched in BAT relative to WAT and other tissues, including mitochondria-rich muscle, suggesting that tissue mitochondrial content is not a determinant of *Letmd1* gene expression (Fig. 1c). We further investigated the temporal regulation of *Letmd1* gene expression in mice and found that *Letmd1* transcript and protein levels increased during the early postnatal period, when thermogenic demand is known to be high (Fig. 1d, e).

We next compared *Letmd1* gene expression in BAT from mice housed at thermoneutrality (30 °C), room temperature (23 °C), or cold (6 °C) conditions and observed an inverse relationship between *Letmd1* gene expression and ambient temperature (Fig. 1f, g). To further investigate the regulation of LETMD1 expression, we intraperitoneally injected mice with CL316,243, a selective β3-adrenoreceptor agonist, and found that LETMD1 protein levels increased in the BAT of CL316,243-treated mice compared to controls (Supplementary Fig. 3). Notably, when cold-exposed mice were re-acclimated to room temperature (Fig. 1h, i), cold-induced expression of *Letmd1* mRNA in BAT reverted to room temperature levels (Fig. 1j), a pattern also seen for the expression of *Ucp1* mRNA (Fig. 1k). Taken together, LETMD1 is enriched in mature brown adipocytes and exhibits dynamic regulation as a function of external cues determining the physiological demand for BAT thermogenesis.

### LETMD1 is a mitochondrial matrix protein

**Fig. 2:**
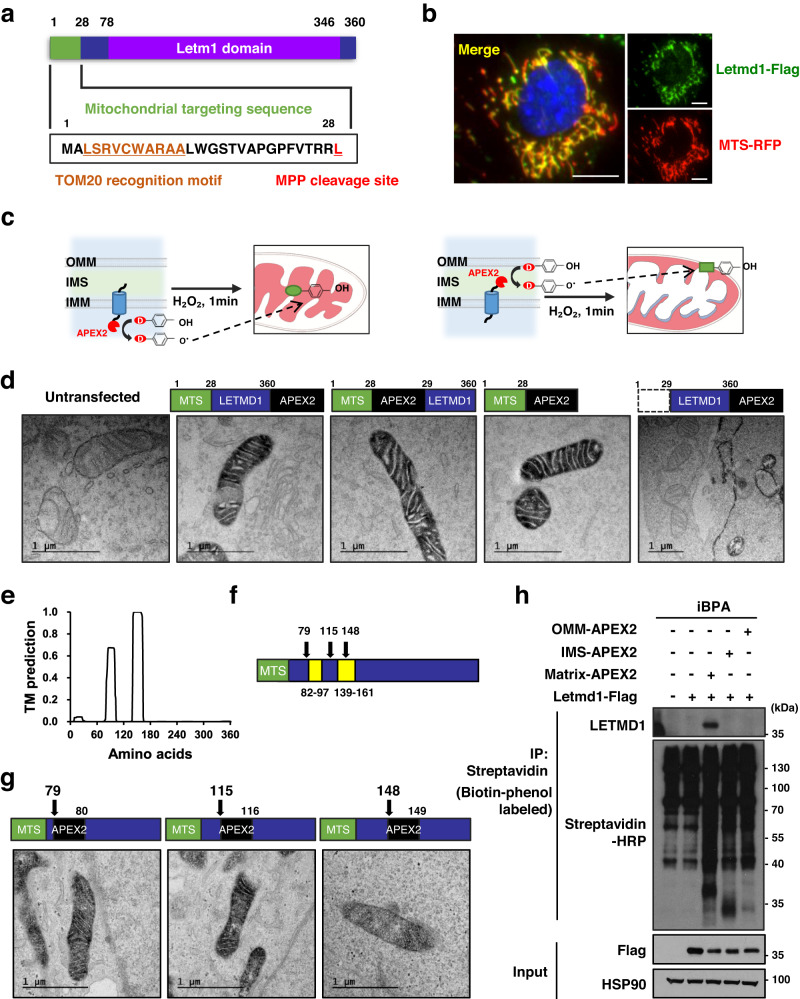
LETMD1 protein is localized to the mitochondrial matrix. **a** Prediction of a putative mitochondrial targeting sequence (MTS) and cleavage site for mouse LETMD1 with MitoFates analysis. **b** Immunofluorescence image of Letmd1-Flag (Green), mitochondria (Red), and DAPI (Blue) in iBPA cells. Representative images from three independent repeats. Scale bar, 10 μm. **c** Experimental scheme of proximity labeling using APEX2 localized to the mitochondrial matrix (left) or intermembrane space (right). APEX2 enzyme (red) labels compartment-specific proteomes using desthiobiotin-phenol (D, DBP) as a substrate. Outer mitochondrial membrane, OMM; Intermembrane space, IMS; Inner mitochondrial membrane, IMM. **d** Electron micrographs of APEX2 staining patterns in untransfected HEK 293 cells and HEK 293 cells transfected with MTS-Letmd1-APEX2 (C-terminus fusion), MTS-APEX2-Letmd1 (N-terminus fusion), MTS-APEX2, and ∆MTS-Letmd1-APEX2 expression constructs. Representative images from two independent repeats. Scale bar, 1 μm. **e** TMHMM analysis of human LETMD1 protein sequence predicting two transmembrane domains (TM). **f** Position of amino acids fusing APEX2 with LETMD1 protein (black arrow). **g** Electron micrographs of APEX2 staining patterns in HEK 293 cells transfected with Letmd1(aa1 ~ 79)-APEX2-Letmd1(aa80 ~ ), Letmd1(aa1 ~ 115)-APEX2-Letmd1(aa116 ~ ) and Letmd1(aa1 ~ 148)-APEX2-Letmd1(aa149 ~ ) expression constructs. Representative images from two independent repeats. Scale bar, 1 μm. **h** Western blots of Flag-tagged LETMD1 protein and OMM-APEX2, IMS-APEX2, and matrix-APEX2 biotinylated proteins in mature brown adipocytes. Representative images from three independent repeats. Input is 3% of the total lysate prior to affinity purification of biotinylated protein species by streptavidin-beads. HSP90 is a loading control. Source data are provided as a Source Data file.

Next, we investigated the subcellular localization of LETMD1 protein in brown adipocytes. Sequence analysis with MitoFates^20^ predicted a putative mitochondrial targeting sequence (MTS) (1-28aa) and a cleavage site by mitochondrial processing peptidases (MPP) (Fig. 2a). In agreement with sequence analysis, immunofluorescence imaging of LETMD1 overexpressing iBPA cells displayed a mitochondrial pattern of localization (Fig. 2b).

To gain more information on the domain structure and cellular localization of LETMD1 protein, we analyzed the activity of a series of APEX2 fusion expression constructs. APEX2 is an engineered peroxidase whose subcellular localization is visualized by electron microscopy (EM) of staining patterns generated by peroxidase activity^21^. High-resolution details offered by EM analysis of APEX2 staining patterns have been successful in determining mitochondrial compartment-specific localization and protein domain topology^22–25^ (Fig. 2c). EM imaging of HEK293 cells transfected with Letmd1-APEX2 (C-terminus APEX fusion) and APEX2-Letmd1 (N-terminus fusion) expression constructs both showed matrix staining patterns indicating that the N-terminus and C-terminus of LETMD1 are exposed to the matrix (Fig. 2d). While we observed a typical mitochondrial matrix pattern of peroxidase staining in cells expressing MTS-APEX2, APEX2 with an N-terminus MTS, cells expressing the Letmd1-APEX2 construct lacking the predicted MTS (1-28aa) abolished mitochondrial localization demonstrating the functionality of the LETMD1 MTS (Fig. 2d).

To further investigate the topological configuration of the LETMD1 protein, we performed TMHMM analysis^26^ which revealed two putative transmembrane domains (82-97aa and 139-161aa) within LETMD1 (Fig. 2e). To experimentally confirm the presence of these predicted transmembrane domains for LETMD1 within the context of a living cell, we analyzed three additional LETMD1-APEX fusion proteins in which APEX was positioned internally at different sites (F79, L115, P148) within LETMD1 (Fig. 2f). Contrary to the predictions from TMHMM analysis, all three internal LETMD1-APEX fusion proteins showed a matrix pattern by EM analysis (Fig. 2g). In another approach, we tested whether LETMD1 protein can be labeled by APEX2 localized to compartments of the mitochondria other than the matrix. APEX2 was expressed in the matrix, intermembrane space (IMS), outer membrane of mitochondria (OMM) compartments and the co-expressed LETMD1-Flag protein was tested for proximity labeling by each mitochondrial compartment-specific APEX. The results showed that the LETMD1-Flag protein was labeled by matrix-APEX but not by OMM-APEX or IMS-APEX (Fig. 2h). Taken together, these results strongly suggest that endogenous LETMD1 protein is a soluble protein residing in the matrix compartment of mitochondria and does not possess a transmembrane domain in its native folded state.

### LETMD1 is required for adaptive thermogenesis

**Fig. 3:**
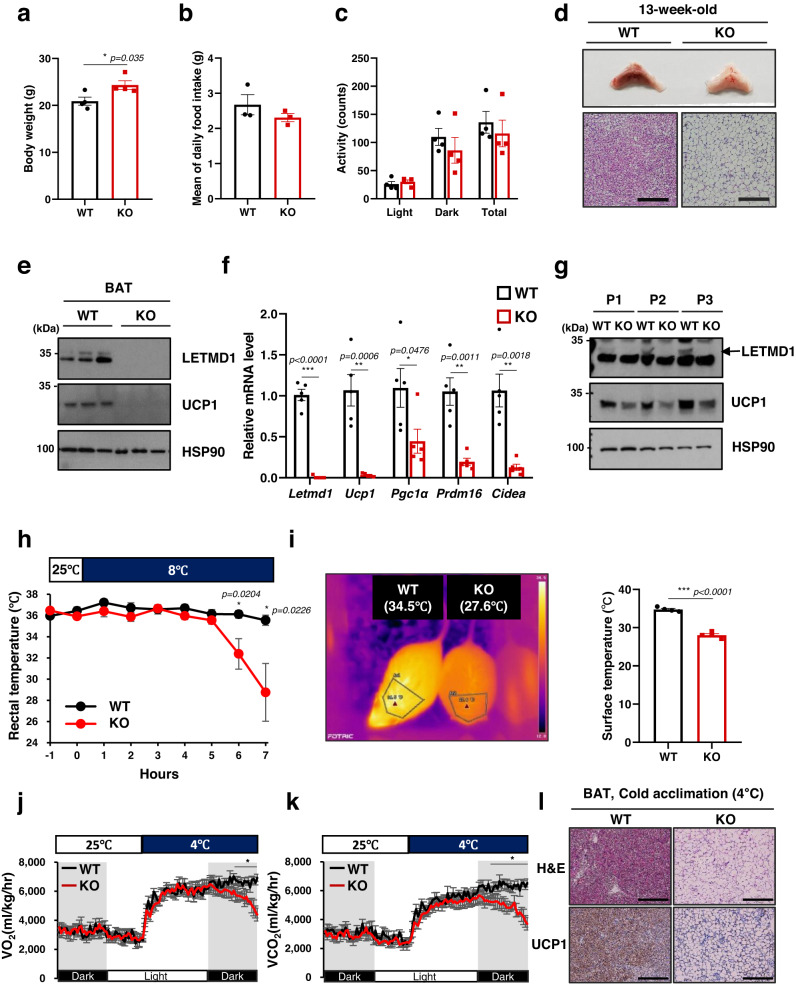
LETMD1 is necessary for adaptive thermogenesis. Body weight (**a**), food intake (**b**), and locomotor activity, (**c**) of 13-week-old wild-type (WT) and *Letmd1* KO (KO) male mice. n_body weight_ = 4, n_food intake_ = 3, n_activity_ = 4. **d** Gross image of brown adipose tissue (BAT) and H&E stained BAT tissue sections from 13-week-old WT and *Letmd1* KO male mice. Representative images from five independent repeats. Scale bar, 200 μm. **e** Western blots of LETMD1 and UCP1 proteins in BAT of 13-week-old male mice. HSP90 is a loading control. **f** mRNA expression of *Letmd1* and brown adipocyte marker genes in the same sets as in (**e**). Expression is normalized to Rpl32. *n* = 5 per group. **g** Western blots of LETMD1 and UCP1 proteins in BAT from male mice on postnatal day 1 (P1), day 2 (P2), and day 3 (P3). HSP90 is loading control. Representative images from three independent repeats. **h** Core body temperature of adult male mice during cold (8 °C) challenge. *n* = 6 per group. **i** Images of surface temperature using infrared thermography after 5 h cold challenge. Images were quantified for the region of interest corresponding to interscapular BAT. *n* = 4 per group. Oxygen consumption rates (**j**) and carbon dioxide production (**k**) rates of WT and *Letmd1* KO male mice exposed to cold (4°C). *n* = 5 per group. **l** Representative H&E staining and UCP1 protein immunostaining of BAT from WT and *Letmd1* KO male mice exposed to cold (4 °C) for 5 h. Representative images from five independent repeats. Scale bar, 200 μm. Data presented as mean ± SEM. *p < 0.05, **p < 0.005, ***p < 0.0005. The significance of the results was assessed using a two-tailed Student’ *t* test. Source data are provided as a Source Data file.

To study the in vivo function of LETMD1, we generated *Letmd1* knockout (KO) mice that are homozygous for a *Letmd1* null allele. Compared to control mice, *Letmd1* KO mice had increased body weight (Fig. 3a) in the absence of significant changes in food intake or physical activity (Fig. 3b, c). Gross examination of *Letmd1* KO BAT revealed a striking whitened appearance and histological analysis revealed dramatically enlarged lipid droplets in *Letmd1* KO BAT compared to littermate controls (Fig. 3d). Furthermore, the expression of *Ucp1* and thermogenic genes was markedly reduced in *Letmd1* KO BAT from adult mice (Fig. 3e, f). This reduction in UCP1 protein levels in *Letmd1* KO BAT was observed immediately after birth and persisted through the perinatal period, when thermogenic demand first emerges (Fig. 3g). These observations led us to examine whether the thermogenic function of BAT is altered in *Letmd1* KO mice. Indeed, we found that *Letmd1* KO mice failed to maintain core body temperature during a cold challenge, indicating a defect in adaptive thermogenesis (Fig. 3h). Infrared imaging of *Letmd1* KO mice further confirmed the inability of *Letmd1* KO mice to activate BAT thermogenesis and maintain body temperature during a cold challenge (Fig. 3i). Although LETMD1 protein was induced in BAT during perinatal stages when thermogenic BAT is highly active, we did not observe any effect on neonatal survival rates due to LETMD1 deficiency under standard housing conditions (Supplementary Fig. 4).

To further characterize physiological alterations in *Letmd1* KO mice, we performed indirect calorimetry of control and *Letmd1* KO mice during a cold challenge. At 25 °C, we did not observe any difference in oxygen consumption (VO_2_) and carbon dioxide production (VCO_2_) between the groups. However, although both wild-type and *Letmd1* KO mice initially increased VO_2_ and VCO_2_ in response to a cold challenge, *Letmd1* KO mice failed to sustain elevated levels of VO_2_ and VCO_2_ which is necessary for a prolonged thermogenic response (Fig. 3j, k). This impaired response to cold stress in *Letmd1* KO mice was accompanied by a failure to upregulate UCP1 protein in BAT tissue (Fig. 3l). These results demonstrate an obligate in vivo requirement for LETMD1 function in the adaptive thermogenic response of BAT upon a cold challenge.

### LETMD1 is essential for BAT mitochondrial integrity and function

**Fig. 4:**
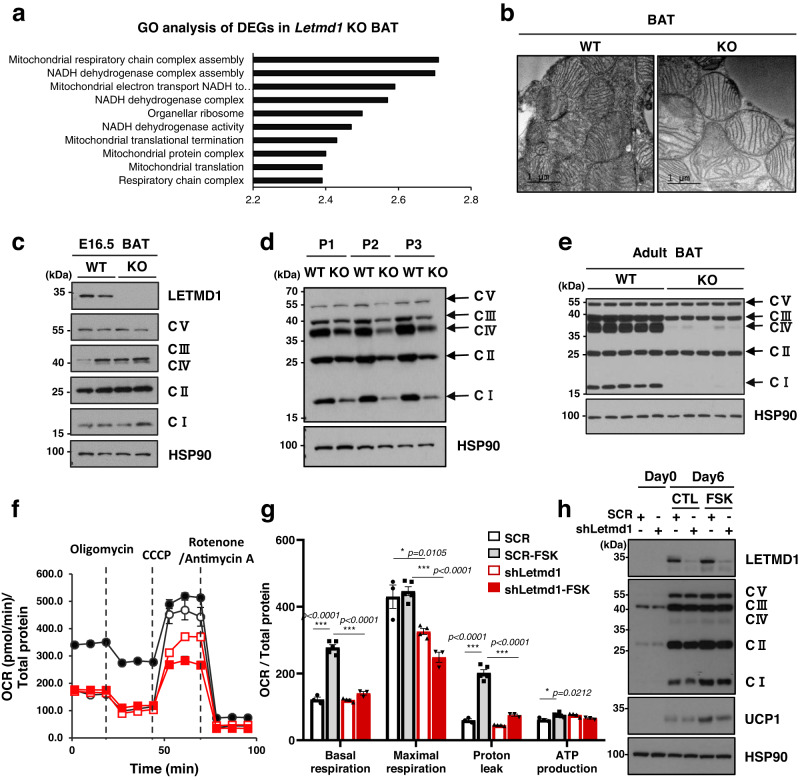
LETMD1 supports mitochondrial respiration of stimulated brown adipocytes. **a** Gene ontology analysis of BAT transcriptomes from wild-type (WT) and *Letmd1* KO (KO) male mice. *n* = 3 per group. **b** Electron micrographs of BAT showing mitochondrial structure. Scale bar, 1 μm. Representative images from three independent repeats.**c** Western blots of OXPHOS complex and LETMD1 proteins from E16.5 embryonic BAT. Representative images from two independent repeats. HSP90 is a loading control. **d** Western blots of OXPHOS complex proteins from perinatal BAT from WT and *Letmd1* KO male mice. Representative images from three independent repeats. HSP90 is a loading control. **e** Western blots of OXPHOS complex proteins from BAT from adult WT and *Letmd1* KO male mice. Representative images from five independent repeats. HSP90 is a loading control. **f** Oxygen consumption rates of control (SCR) and *Letmd1* knockdown (shLetmd1) brown adipocytes in response to 4 h pre-treatment of 10 µM forskolin (FSK). n_SCR_ = 3, n_SCR-FSK_ = 5, n_shLetmd1_ = 4, n_shLetmd1-FSK_ = 3. **g** Basal respiration, maximal respiration, proton leak, and ATP production in the same sets as (**f**). The OCR values were normalized to the protein levels of each well. Data presented as mean ± SEM. **h** Western blots of LETMD1, OXPHOS complex, and UCP1 proteins in the same sets as (**f**). Representative images from three independent repeats. *p < 0.05, **p < 0.005, ***p < 0.0005. The significance of the results was assessed using one-way ANOVA. Source data are provided as a Source Data file.

To investigate the molecular role of LETMD1 in brown adipocyte function and adaptive thermogenesis, we performed RNA-seq analysis of *Letmd1* KO and wild-type BAT. Gene ontology (GO) analysis of transcriptome changes revealed down-regulation of a broad array of genes involved in processes that occur in the mitochondrial matrix compartment in *Letmd1* KO BAT (Fig. 4a). At the ultrastructural level, *Letmd1* KO BAT contained dysmorphic mitochondria with sparse distended cristae, a principal site of OXPHOS complex (Fig. 4b). These data led us to directly examine OXPHOS complex components and functions in *Letmd1* KO BAT. At embryonic day 16.5, when thermogenic demand is absent, BAT OXPHOS complex proteins were unaffected by LETMD1 deficiency (Fig. 4c). However, during the early postnatal period, control BAT upregulated OXPHOS complex proteins in response to thermogenic demand but *Letmd1* KO BAT failed to upregulate OXPHOS proteins during this critical period for BAT thermogenesis (Fig. 4d). The defective OXPHOS protein expression in *Letmd1* KO BAT was further exacerbated in adult stages and showed a near complete loss of complex I and IV proteins (Fig. 4e).

To examine the functional consequence of OXPHOS complex deficiency in *Letmd1* KO BAT, we measured respiratory function in scrambled control (SCR) and *Letmd1* knockdown brown adipocytes (shLetmd1) in the presence or absence of forskolin stimulation. We found that there were no significant differences in basal respiration between control (SCR) and shLetmd1 brown adipocytes in the unstimulated state (Fig. 4f, g). However, upon forskolin (FSK) stimulation, we observed a dramatic reduction in both basal and maximal respiration of shLetmd1 cells, in contrast to control (SCR) cells (Fig. 4f, g). In forskolin-stimulated conditions, the proton leak of *Letmd1* knockdown cells was also significantly reduced, indicating that *Letmd1* knockdown leads to impaired UCP1 activity (Fig. 4g). Analysis of OXPHOS and UCP1 expression in *Letmd1* knockdown cells showed a slight decrease in OXPHOS complex I and IV, as well as UCP1 protein levels (Fig. 4h). Additional experiments showed that *Letmd1* knockdown impairs mitochondrial respiration and thermogenesis in brown adipocytes stimulated by isoproterenol (Supplementary Fig. 5a, b). Conversely, *Ucp1* knockdown had no effect on either *Letmd1* mRNA or protein expression levels but did result in decreased expression of OXPHOS components (Supplementary Fig. 6). These findings suggest that LETMD1 functions upstream of UCP1 in regulating mitochondrial function in brown adipocytes. Taken together, these results demonstrate the crucial role of LETMD1 in supporting brown adipocyte OXPHOS function in a stimulation-dependent manner.

### LETMD1 functions in a BAT-autonomous manner

**Fig. 5:**
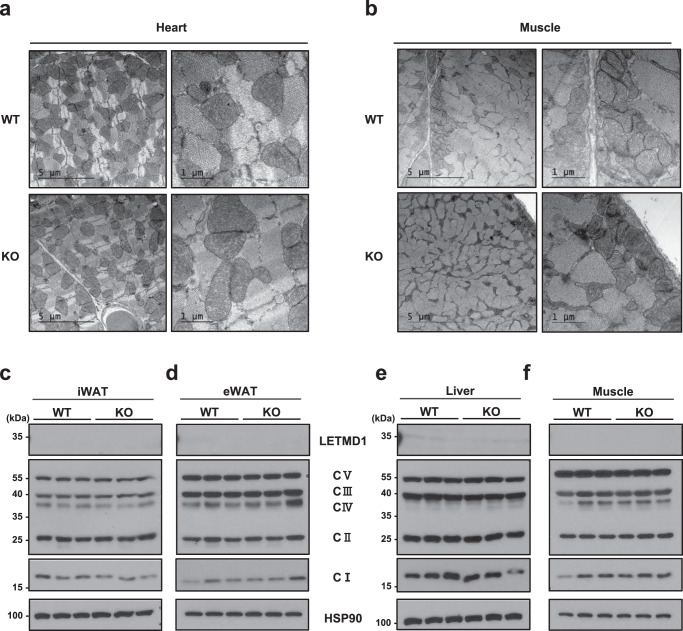
LETMD1 deficiency does not affect mitochondrial ultrastructure or OXPHOS complex expression in tissues other than BAT. Electron micrographs of mitochondria from heart (**a**) and soleus muscle (**b**) of adult wild-type (WT) and *Letmd1* KO (KO) male mice. Representative images from three independent repeats. Scale bar, 5 μm and 1 μm respectively. Western blots were performed to evaluate mitochondrial OXPHOS complex expression in inguinal white adipose tissue (iWAT) (**c**), epididymal white adipose tissue (eWAT) (**d**), liver (**e**), and gastrocnemius muscle (**f**) from 13-week-old male WT and *Letmd1* KO mice. *n* = 3 per group. HSP90 is a loading control. Representative images from three independent repeats. Source data are provided as a Source Data file.

**Fig. 6:**
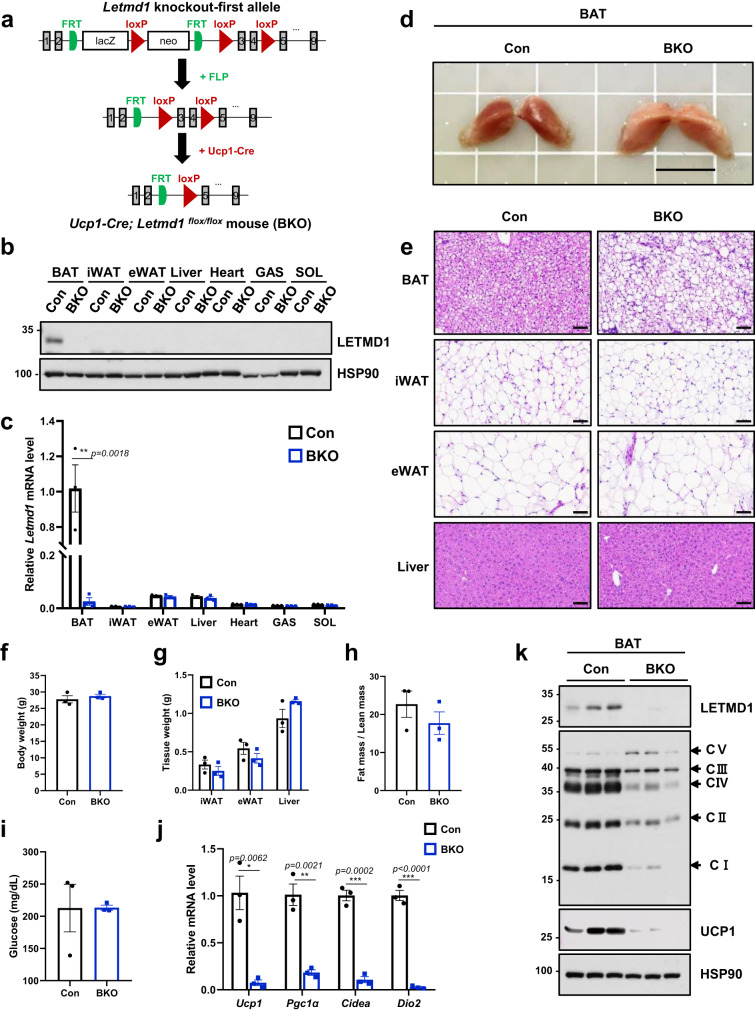
Mice with BAT-specific deletion of *Letmd1* have abnormal brown fat and reduced OXPHOS complex expression similar to *Letmd1* KO mice. **a** Strategy for generating *Letmd1* conditional alleles. See “Materials and Methods” for details. LETMD1 protein (**b**) and mRNA (**c**) expression levels were measured in tissues (BAT brown adipose tissue, iWAT inguinal white adipose tissue, eWAT epididymal white adipose tissue, liver, heart, GAS gastrocnemius muscle, SOL soleus muscle) from 12-week-old male *Letmd1*^*flox/flox*^ mice (Con) and *Ucp1-Cre; Letmd1*^*flox/flox*^ mice (BKO). *n* = 3 per group. Representative results from three independent repeats. **d** Gross images of brown adipose tissue from 12-week-old male *Letmd1*^*flox/flox*^ mice (Con) and Ucp1-Cre; *Letmd1*^*flox/flox*^ mice (BKO). Scale bar, 1 cm. Representative images from three independent repeats. **e** Representative hematoxylin and eosin (H&E) staining of BAT, iWAT, eWAT, and liver from 12-week-old male *Letmd1*^*flox/flox*^ mice (Con) and *Ucp1-Cre; Letmd1*^*flox/flox*^ mice (BKO). Scale bar, 60 µm. Representative images from three independent repeats. Body weight (**f**), tissue weight (**g**), fat mass/lean mass (**h**), and blood glucose levels (**i**) of 12-week-old male *Letmd1*^*flox/flox*^ mice (Con) and *Ucp1-Cre; Letmd*^*flox/flox*^ mice (BKO). *n* = 3 per group. **j** mRNA expression of thermogenic genes. mRNA expression is normalized to Rpl32. *n* = 3 per group. Data presented as mean ± SEM. *p < 0.05, **p < 0.005, ***p < 0.0005 **k** Western blots of LETMD1 protein, mitochondrial complex components, and UCP1 protein in the BAT from 12-week-old male *Letmd*^*flox/flox*^ mice (Con) and *Ucp1-Cre; Letmd1*^*flox/flox*^ mice (BKO). HSP90 is loading control. Representative images from three independent repeats. The significance of the results was assessed using a two-tailed Student’ *t* test. Source data are provided as a Source Data file.

We next examined whether the structural and functional defects observed in *Letmd1* KO mice were specific to BAT. To this end, we analyzed mitochondrial ultrastructure in metabolic tissues with high mitochondrial content such as heart and soleus muscle, and found no significant differences between *Letmd1* KO and control (Fig. 5a, b). We also quantified OXPHOS complex proteins in metabolic tissues including iWAT, eWAT, liver, and muscle tissue lysates by western blot. In contrast to the reduced OXPHOS complex levels observed in BAT, the results show that other *Letmd1* KO tissues have normal levels of OXPHOS complex expression (Fig. 5c–f). To thoroughly investigate the specific requirements of LETMD1 in BAT, we generated mice with a *Letmd1* conditional knockout allele and crossed them with *Ucp1-Cre* mice to obtain *Ucp1-Cre; Letmd1*^*flox/flox*^ (*Letmd1* BKO) (Fig. 6a), which were confirmed to have BAT-specific deletion of *Letmd1* (Fig. 6b, c). Similar to *Letmd1* KO BAT, gross examination of BAT from *Letmd1* BKO BAT showed a whitened appearance (Fig. 6d) and histological analysis of BAT showed brown adipocytes with enlarged lipid droplets (Fig. 6e). There were no differences in body weight (Fig. 6f), fat and liver tissue weights (Fig. 6g), fat mass/lean mass ratios (Fig. 6h), and fasting blood glucose (Fig. 6i) between *Letmd1* BKO and control mice. However, the key molecular phenotypes observed in *Letmd1* KO mice such as mRNA expression of BAT thermogenic genes (Fig. 6j) and reduced expression of OXPHOS complex proteins and UCP1 (Fig. 6k) were recapitulated in *Letmd1* BKO mice. These results strongly support the notion that LETMD1 plays a tissue-autonomous role that is specific to BAT mitochondria function and thermogenesis.

## Discussion

Adaptive thermogenesis of BAT is critical for defending against cold stress and maintaining body temperature in mammals. A mitochondrial protein, UCP1, plays a key role in this process by uncoupling ATP production from the proton motive force generated by OXPHOS complex. However, additional mitochondrial factors required for BAT thermogenesis are largely unknown. In this study, we uncover an essential role for the BAT-enriched protein LETMD1 in mitochondrial respiration and adaptive thermogenesis.

LETMD1 is annotated as a mitochondrial protein in MitoCarta 1.0^27^, MitoCarta 2.0^28^ and MitoCarta 3.0^29^. Furthermore, LETMD1 was categorized as one of the mitochondrial uncharacterized proteins (MXPs) in humans^30^ but its specific molecular and physiological function in BAT has not been explored. Here, we demonstrate that LETMD1 has critical functions in regulating mitochondrial which is essential for maintaining BAT activation during adaptive thermogenesis.

Our proximity labeling enzyme-assisted topology studies demonstrate that LETMD1 is a mitochondrial matrix protein, which contradicts a previous study suggesting LETMD1 protein as an outer mitochondrial membrane protein^31^ (Q924L1, UniProt). In our experiments, LETMD1 proteins fused with APEX revealed that LETMD1 protein lacks transmembrane domains, and N and C termini are exposed to the mitochondrial matrix (Fig. 2). In *Letmd1* knockdown iBPA brown adipocytes, LETMD1-APEX2 fusion protein restored UCP1 expression functionally like LETMD1 protein (Supplementary Fig. 7). While APEX insertion is unlikely to cause misfolding, it cannot be completely excluded. Despite these concerns, Letmd1-APEX proteins with APEX insertion demonstrate both catalytic activity and matrix-targeted localization, suggesting that both Letmd1 and APEX are properly folded in the Letmd1-APEX fusion protein (Fig. 2g).

Our results also reveal significant differences in topology between LETMD1 protein and the related LETM1. Unlike LETM1, which is reported to have two transmembrane domains and both termini are in the mitochondrial matrix^25^, our results indicate that LETMD1 has a fully matrix-localized topology that suggests its role in mitochondrial function may differ from that claimed for LETM1. Despite these differences, LETMD1 and LETM1 function show similarities as LETM1 regulates mitochondrial swelling^32^, assembly of the respiratory chains^33^, and cristae organization^34^. Several studies have reported that LETM1 (SLC55A1) functions as a K^+^/H^+^ exchanger (KHE)^35–37^ or Ca^2+^/H^+^ antiporter (CHE)^38–41^. However, it is important to note that the proposed topology of these LETM1 and LETMD1 were derived from different analyses, and additional investigations will be required to comprehensively evaluate the functional and mechanistic differences between these two proteins.

The mitochondrial matrix is the key locale for coordinating the activity of the OXPHOS complex and UCP1 protein-mediated uncoupling, which together maintain the thermogenic program of brown adipocytes. We observed a slight decrease in OXPHOS complex I and IV associated with *Letmd1* knockdown in cultured brown adipocytes (Fig. 4h), but this difference was not as pronounced as what was seen in the adult BAT of *Letmd1* KO mice (Fig. 4e). These results suggest that the severely impaired OCR response in brown adipocytes by *Letmd1* knockdown under forskolin or isoproterenol-treated conditions cannot be fully explained by decreased expression of OXPHOS complex proteins. Nevertheless, we consistently observed pronounced reduction of OXPHOS complex I and IV protein in *Letmd1* KO BAT which would contribute to impaired BAT thermogenesis upon acute cold challenge (Fig. 3h, j). The reason for the difference between the effects of LETMD1 deficiency on OXPHOS complex protein levels in in vivo BAT versus in vitro brown adipocytes is an important topic for future research.

A notable observation from previous studies is that *Ucp1* KO mice exhibit a marked decrease in OXPHOS complex expression, indicating a unique interdependence between UCP1 protein and OXPHOS function in BAT^42^. In the setting of UCP1 deficiency, cold-induced activation of BAT metabolism leads to a ~ 95% decrease in OXPHOS complex I and IV subunits. The study also demonstrates that environmental cold stimulus, rather than innate abnormalities in BAT development, is responsible for the diminished expression of electron transport chain components in *Ucp1 KO* mice^42^, which parallels the phenotype we observed in the BAT of *Letmd1* KO mice (Figs. 3 and 4). In our study, we have demonstrated that UCP1 protein levels decrease in the BAT of *Letmd1* KO mice and *Letmd1* knockdown brown adipocytes (Fig. 3g and 4h), while a global proteomics analysis by Spiegelman and colleagues indicates that *Ucp1* KO has no effect on LETMD1 protein levels^42^. Interestingly, we also found that *Ucp1* knockdown in differentiated iBPA brown adipocytes had no effect on LETMD1 expression, but did lead to decreased OXPHOS protein levels (Supplementary Fig. 6). These results provide strong evidence that LETMD1 acts upstream of UCP1, although the precise mechanism through which LETMD1 regulates UCP1 expression remains unclear and warrants further investigation. However, we propose that the decrease in OXPHOS complex levels, particularly complex I and IV, in *Letmd1* KO BAT is likely due to the downregulation of UCP1. Furthermore, we find that the observed effect of LETMD1 deficiency on OXPHOS complex levels is specific to BAT (Figs. 4 and 5), thereby highlighting a BAT-specific and BAT-autonomous role for LETMD1 in mitochondrial OXPHOS and uncoupled respiration. Finally, our demonstration that mice with a BAT-specific deletion of *Letmd1* exhibit phenotypes identical to those observed in whole-body *Letmd1* KO mice provides additional evidence for the BAT-autonomous requirement of LETMD1 (Fig. 6).

Increasing the activity of BAT in humans has emerged as a promising strategy to prevent obesity and metabolic disorders by enhancing energy expenditure^8,9^. Consequently, the identification of novel molecular regulators of mitochondrial metabolism and BAT thermogenesis presents a significant opportunity for preventive and therapeutic interventions for obesity^43,44^. In this context, we have demonstrated that LETMD1, a mitochondrial matrix protein that is specifically enriched in BAT and dynamically regulated in response to thermogenic stimuli, is a crucial component required for thermogenic BAT function. Moreover, analysis using our Gene-Module Association Determination (G-MAD)^45^ approach reveals that LETMD1 is strongly associated with mitochondrial function in both mouse and human adipose tissues (Supplementary Fig. 8). These findings provide further support for the role of LETMD1 in regulating mitochondrial metabolism and BAT thermogenesis and highlight its potential as a therapeutic target for the treatment of obesity and metabolic diseases.

During the process of revising our manuscript, we came across several recently published reports that have also investigated the role of LETMD1 in BAT function^46–49^. While these studies consistently demonstrated the upregulation of LETMD1 in response to cold stimulation and impaired thermogenic function in *Letmd1* KO mice, each report describes distinct aspects of LETMD1 function. For instance, Choi et al.^46^ demonstrated an interaction between LETMD1 and Brg1 in the nucleus, suggesting a potential link to transcriptional regulation of BAT thermogenesis. Snyder et al.^47^ demonstrated that LETMD1 is essential for BAT structure and OXPHOS expression, even under thermoneutral conditions. Song et al.^48^ observed perturbations in mitochondrial calcium homeostasis in brown adipocytes lacking LETMD1, while Xiao et al.^49^ reported the localization of LETMD1 to the mitochondrial inner membrane. In contrast, our study provides conclusive evidence that LETMD1 functions autonomously within brown adipocytes, as demonstrated by the phenotype of brown adipocyte-specific *Letmd1* KO mice. Moreover, our results show that LETMD1 is a mitochondrial matrix protein using proximity labeling techniques, providing a more accurate representation of LETMD1 protein topology in a live cell. Our findings complement and converge upon the growing body of literature underscoring the critical role of LETMD1 and its function in BAT thermogenesis.

## Methods

### Ethics statement

All animal studies were performed in compliance with the institutional guidelines of the Korean Research Institute of Biotechnology and Bioscience, and all mouse experiments were approved and performed under institutional guidelines.

### Animal experiments

The ES cell clone 84278, which was generated by the International Knockout Mouse Consortium (IKMC) and contains a knockout-first allele of the *Letmd1* gene, was purchased from The European Conditional Mouse Mutagenesis Program (EUCOMM). The *Letmd1* knockout-first targeting vector contains a *lacZ* and a neomycin resistance gene cassette that is flanked by Flp recombinase target (FRT) sequences. This cassette was inserted into an intron between exon 2 and exon 3 of *Letmd1* gene to disrupt endogenous LETMD1 expression. Exon 3 and exon 4 of *Letmd1* gene are also flanked by loxP sites and there is a loxP site between the lacZ and neomycin resistance gene. *Letmd1* heterozygous null mice (*Letmd1 +/−*) were obtained by microinjection of this ES cell clone into C57BL6/N mice (Macrogen). To generate *Letmd1* conditional knockout mice that harbor a *Letmd1* floxed allele, we bred mice with the *Letmd1* knockout-first allele with ACTB-Flpe mice (JAX 003800). Mice containing the *Letmd1* floxed allele were further bred to *Ucp1-Cre* mice (JAX 024670) to generate mice with BAT-specific deletion of the *Letmd1* gene (*Ucp1-Cre; Letmd*^*flox/flox*^). Unless otherwise specified, all experiments involving mice were conducted using male mice between 12 and 17 weeks of age. Mice were maintained in a specific pathogen-free animal facility under institutional guidelines of the Korean Research Institute of Biotechnology and Bioscience.

### Cell culture, adipogenic differentiation, and Oil-Red-O staining

An immortalized brown preadipocyte (iBPA) cell line was kindly provided by Dr. Shingo Kajimura (UCSF, San Francisco, CA, USA) and grown in high glucose Dulbecco’s modified Eagle medium (DMEM, Gibco) containing 10% fetal bovine serum (Gibco) and 1% antibiotics at 37 °C in a humidified atmosphere with 5% CO_2_. For brown adipocyte differentiation, cells were induced as previously described^19^. In brief, cells were cultured to confluence (day 0) in a maintenance medium containing 10% FBS, and 1% antibiotics, and then induced to differentiate in a differentiation medium (DMEM containing 10% FBS) supplemented with 0.5 mM isobutylmethylxanthine (IBMX, Sigma I5879), 0.5 µM dexamethasone (Sigma D1756), 20 nM insulin (Santa cruz sc-360248), 1 nM 3,3′,5-Triiodo-L-thyronine (T3, Sigma T2877), and 125 µM indomethacin (Sigma I7378) for 2 days, after which (day 2) the cells were further induced to differentiate and maturate for 4 days in the growth medium composed of DMEM,10% FBS, 20 nM insulin and 1 nM T3.

### Generation of stable overexpression or knockdown cell lines

To construct iBPA cells stably expressing FLAG-tagged mouse LETMD1, a retroviral infection system was used. For LETMD1 expression, DNA encoding the FLAG-tagged Letmd1 was inserted into the pRetroX-IRES-ZsGreen1 vector (Clontech Laboratories, Mountain View, CA, USA). For virus production, GP2-293 packaging cells (Clontech, 631458) were transfected using TransIT®-LT1 Transfection Reagent (Mirus, MIR2300), and infected cells were selected using a FACSAria cell sorter (BD Biosciences) and maintained in high glucose Dulbecco’s modified Eagle medium (DMEM) containing 10% fetal bovine serum (Gibco). To knockdown endogenous LETMD1 expression, we used a retrovirus-mediated shRNA system. Short-hairpin shRNAs were designed by selecting a target sequence for the mouse *Letmd1* gene according to Knockout RNAi systems user manual (Clontech). The shRNA against *Letmd1* was inserted into the multi-cloning site of the pSIREN-RetroQ-DsRed vector (Clontech). The following shRNA sequences targeting the coding region of *Letmd1* mRNA were used to knockdown *Letmd1* expression: 5ʹ- GATCCGCAACTGCTAGTCAAGCATTTCAAGAGAATGCTTGACTAGCAGTTGCTTTTTTG-3ʹ and 5ʹ- AATTCAAAAAAGCAACTGCTAGTCAAGCATTCTCTTGAAATGCTTGACTAGCAGTTGCG-3ʹ. Control shRNA (scrambled) vector was provided by Clontech.

### Indirect calorimetry

For indirect calorimetry studies, mice were housed individually in metabolic cages (CLAMS12, Columbus Instruments) with ad libitum access to food and water. Oxygen consumption rates and carbon dioxide production rates were measured for 48 h. The activity was monitored simultaneously with metabolic measurements.

### Core body temperature measurement and Infrared camera imaging

For measuring core temperature of cold exposed mice, mice were exposed to 8 °C in slow-temperature chamber (DHIN02-0034, DBL) and core body temperature was monitored using a rectal thermometer (Testo 925, Testo). The surface temperature of mice was measured with an infrared camera (Fortric 228, Fortric precision instruments) and analyzed with AnalyzIR (Fortric precision instruments) software.

### Histology analysis

Mouse tissues were fixed in 10% neutral buffered formalin (Sigma, HT501128) for 24 h and embedded in paraffin by an automated tissue processor (Leica, TP1020). In total, 4 μm-thick tissue sections were obtained, deparaffinized, rehydrated, and stained with hematoxylin and eosin.

### Extracellular flux assays

Oxygen consumption rate (OCR) was measured using the XFe96 extracellular flux analyzer (Seahorse Bioscience). On the day before the experiment, the sensor cartridge was placed into the calibration buffer (Seahorse Bioscience) and incubated at 37 °C in a non-CO_2_ incubator. Prior to measurement, cells were equilibrated in an assay medium containing 25 mM D-glucose, 4 mM L-glutamine, and 1 mM sodium pyruvate for 1 h. To measure OCR by mitochondrial respiration, cells were treated sequentially with 2.5 µM oligomycin, 5 µM Carbonyl cyanide m-chlorophenyl hydrazone (CCCP), and 2 µM rotenone/5 µM antimycin A. OCR was measured 4 h after 10 µM forskolin or 1 h after 1 µM isoproterenol treatment. At the end of each assay, cells were lysed with ice-cold RIPA buffer, and the protein contents were measured by Bradford assay. The OCR values were normalized to the protein levels of each well. The data were analyzed using Wave 2.6.1 software (Agilent).

### RNA sequencing

Total RNA was isolated using Trizol reagent (Invitrogen). RNA quality was assessed by Agilent 2100 bioanalyzer using the RNA 6000 Nano Chip (Agilent Technologies, Amstelveen, The Netherlands), and RNA quantification was performed using ND-2000 Spectrophotometer (Thermo Inc., DE, USA). For control and test RNAs, the construction of library was performed using QuantSeq 3ʹ mRNA-Seq Library Prep Kit (Lexogen, Inc., Austria) according to the manufacturer’s instructions. In brief, each 500 ng total RNA were prepared and an oligo-dT primer containing an Illumina-compatible sequence at its 5ʹ end was hybridized to the RNA and reverse transcription was performed. After degradation of the RNA template, second strand synthesis was initiated by a random primer containing an Illumina-compatible linker sequence at its 5ʹ end. The double-stranded library was purified by using magnetic beads to remove all reaction components. The library was amplified to add the complete adapter sequences required for cluster generation. The finished library is purified from PCR components. High-throughput sequencing was performed as single-end 75 sequencing using NextSeq 500 (Illumina, Inc., USA). QuantSeq 3ʹ mRNA-Seq reads were aligned using Bowtie2^50^. Bowtie2 indices were either generated from genome assembly sequence or the representative transcript sequences for aligning to the genome and transcriptome. The alignment file was used for assembling transcripts, estimating their abundances and detecting differential expression of genes. Differentially expressed gene were determined based on counts from unique and multiple alignments using coverage in Bedtools^51^. The RC (Read Count) data were processed based on quantile normalization method using EdgeR within R^52^ using Bioconductor^53^. Gene classification was based on searches done by DAVID (http://david.abcc.ncifcrf.gov/) and Medline databases (http://www.ncbi.nlm.nih.gov/). Data mining and graphic visualization were performed using ExDEGA (Ebiogen Inc., Korea).

### Biotin-phenol labeling in live cells

APEX2-fusion expression vectors were introduced into HEK 293 T (CRL-3216, ATCC), or iBPA cells for biotin-phenol labeling experiments. Viral expression of either the LETMD1-APEX fusion protein or the wild-type LETMD1 protein in 3ʹ UTR-targeted Letmd1 shRNA knockdown iBPA cells restored *Ucp1* expression to a similar extent, indicating that LETMD1-APEX fusion protein retains the functional properties of the wild-type LETMD1 protein (Supplementary Fig. 7). To prepare iBPA cells for biotin-phenol labeling, we introduced APEX2-fusion expression constructs to iBPA cells through electroporation and induced their differentiation into brown adipocytes. At day 6 of differentiation, the culture medium was replaced with fresh growth medium containing 500 µM desthiobiotin-phenol (DBP), followed by incubation at 37 °C under CO_2_ for 30 min. The cells were then treated with 1 mM H_2_O_2_ and gently agitated for 1 min at room temperature. To terminate the reaction, the cells were washed thrice with ice-cold DPBS containing 5 mM trolox, 10 mM sodium ascorbate, and 10 mM sodium azide. Subsequently, the cells were lysed for western blot analysis or fixed for imaging analysis. We performed western blot analysis, fluorescence microscope imaging, and transmission electron microscope (TEM) imaging of DBP-labeled cells as described previously^54,55^.

### Western blot analysis

Homogenized tissue or cells were lysed with ice-cold RIPA buffer (Pierce, 89900) containing 1X protease inhibitor cocktail (GenDEPOT, P3100) and incubated at 4 °C for 30 min. After centrifugation at 15,000 × *g* for 15 min, the supernatant was moved to a new tube. Protein concentrations were measured using the Bradford assay (Bio-Rad, 5000001). Protein samples were directly analyzed using sodium dodecyl sulfate-polyacrylamide gel electrophoresis (SDS-PAGE) with SDS sample buffer (60 mM Tris-Cl (pH 6.8), 10% sodium lauryl sulfate, 25% glycerol, 100 mM dithiothreitol, 0.04% Bromophenol blue) without boiling. Western blot analysis was performed according to standard methods. Antibodies used in immunoblot analyses included those against LETMD1 (LSBio, LS-C384640, 1:1000); UCP1 (Abcam, ab10983, 1:1000); HSP90 (Santa Cruz, sc-7947, 1:2000); α-Tubulin (Sigma-Aldrich, sc-8035, 1:3000); OXPHOS cocktail (Abcam, ab110413, 1:1000); Streptavidin-HRP (Thermo Scientific, S911, 1:3000); and Flag (Sigma-Aldrich, F3165, 1:3000). The specific signals were amplified by horseradish peroxidase-conjugated secondary anti-rabbit or anti-mouse antibody (Santa Cruz, 1:3000).

### mRNA expression analysis by qRT-PCR

Total RNA was extracted from tissue or cultured cells using TRIzol Reagent (Invitrogen, 15596026) according to manufacturer instructions, and first-strand cDNA was synthesized from total RNA using the reverse transcriptase M-MLV (Promega, M1701) and a random primer (Promega, C1181) according to manufacturer protocols. Real-time PCR was performed using 2X Real-time PCR Smart mix kit (SolGent, SRH91-R500) according to the manufacturer protocols. The data were analyzed using CFX Maestro software 2.0 (Bio-Rad). Gene expression levels were normalized to 60 S ribosomal protein L32 (Rpl32). Primers used for qRT-PCR are listed in Supplementary Table 1.

### Statistics and reproducibility

Statistical analysis was performed using Prism 8.4 software (GraphPad). All statistics are described in figure legends. In general, comparisons between two groups were performed using the two-tailed Student’s *t* test and multiple group comparisons were performed by one-way ANOVA followed by Tukey-Kramer post-hoc test. Data are expressed as mean ± standard error of the mean (SEM). Results with *P* values less than 0.05 were considered statistically significant.

### Reporting summary

Further information on research design is available in the Nature Portfolio Reporting Summary linked to this article.

## Supplementary information

Supplementary Information

Reporting Summary

## Data Availability

We analyzed publicly available gene expression data for genes that are enriched in BAT, as compared to epididymal WAT (eWAT) (GSE92844) and induced by cold stimulus (GSE70437). RNA sequencing data are deposited in Gene Expression Omnibus (GEO) under accession number GSE232223. The source data for this study are provided with this paper. Source data are provided with this paper.

## Source data

Source Data
