# Supplementary material for: Mitochondrial matrix protein LETMD1 maintains thermogenic capacity of brown adipose tissue in male mice
Source: Nat Commun. 2023 Jun 23;14:3746. doi: 10.1038/s41467-023-39106-z (PMC10290150; doi:10.1038/s41467-023-39106-z)

Supplementary Information

Mitochondrial matrix protein LETMD1 maintains thermogenic capacity of brown adipose tissue in male mice

Authors

Anna Park, Kwang-eun Kim, Isaac Park, Sang Heon Lee, Kun-Young Park, Minkyo Jung, Xiaoxu Li, Maroun Bou Sleiman, Su Jeong Lee, Dae-Soo Kim, Jaehoon Kim, Dae-Sik Lim, Eui-Jeon Woo, Eun Woo Lee, Baek Soo Han, Kyoung-Jin Oh, Sang Chul Lee, Johan Auwerx, Ji Young Mun, Hyun-Woo Rhee, Won Kon Kim, Kwang-Hee Bae, Jae Myoung Suh

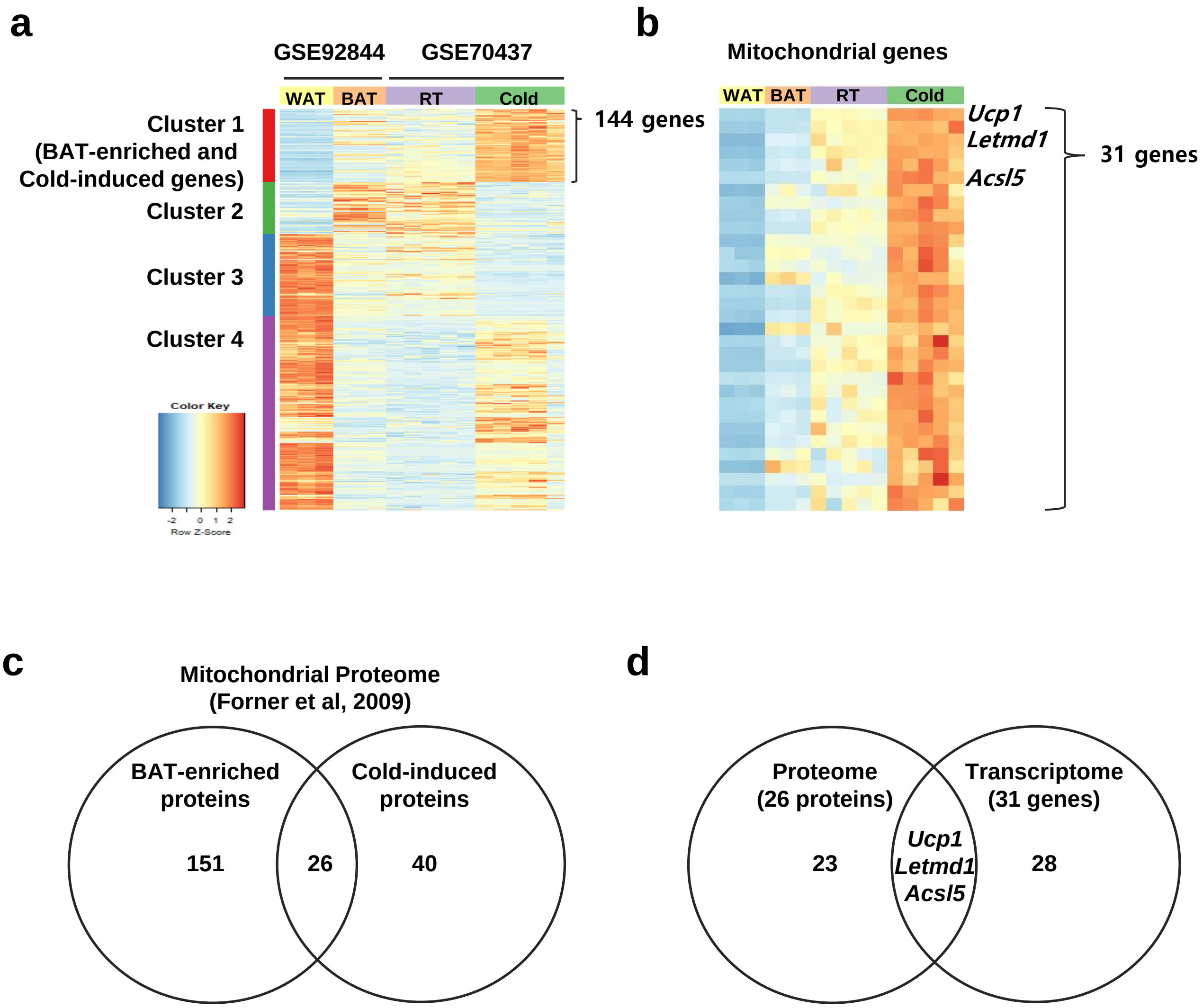

Supplementary Figure 1. Identification of cold-inducible mitochondrial proteins in brown adipose tissue by transcriptome and proteome analysis. a, Cluster analysis of differentially expressed genes in BAT (GSE92844, (Mo et al., 2017) and cold exposed condition (GSE70437, (Marcher et al., 2015)). Cluster 1 (BAT-enriched and Cold-induced genes) was selected for genes that increased or decreased > 1.5 fold (p value < *0.05*) in a combined analysis of two independent RNA sequencing datasets, GSE92844 and GSE70437). **b,** Gene expression heat map of 31 mitochondrial genes identified from cluster 1 based on MitoCarta 2.0 annotations. **c,** Venn diagram showing the number of mitochondrial proteins highly expressed in BAT and cold-exposed BAT in proteomics studies (Forner et al., 2009). Proteome analysis was performed from Table S4 and Table S8 as presented in Forner et al, 2009 paper. 177 proteins in the top 30% of BAT/WAT ratios were selected as BAT-enriched proteins in Table S4. Cold-induced proteins are the 66 proteins annotated in MitoCarta 2.0 with log2 (Acute/CTRL) > 0.5 as listed in Table S8. The overlap between BAT-enriched proteins and cold-induced proteins in BAT resulted in 26 mitochondrial proteins. **d,** Venn diagram showing selected genes from the combined analysis of the transcriptome (b) and proteome analysis (c).

Transcriptome analysis (GSE92844, GSE70437)	
**Gene**	**Description**
**Ucp1**	Uncoupling protein 1 (mitochondrial, proton carrier)
**Gpd1**	Glycerol-3-phosphate dehydrogenase 1 (soluble)
**Letmd1**	LETM1 domain containing 1 (Letmd1)
**Slc25a20**	Solute carrier family 25 (mitochondrial carnitine/acylcarnitine translocase), member 20
**Ddt**	D-dopachrome decarboxylase
**Acsl5**	Long-chain-fatty-acid--CoA ligase 5
**Pdk4**	Pyruvate dehydrogenase kinase, isoenzyme 4
**Slc25a39**	Solute carrier family 25 member 39
**Mcee**	Methylmalonyl-CoA epimerase, mitochondrial
**Letm1**	Mitochondrial proton/calcium exchanger protein
**Dnaja3**	DnaJ homolog subfamily A member 3, mitochondrial
**Fkbp4**	Peptidyl-prolyl cis-trans isomerase FKBP4 Peptidyl-prolyl cis-trans isomerase FKBP4, N-terminally processed
**Yif1b**	Yip1 interacting factor homolog B
**Grsf1**	G-rich sequence factor 1
**Mlycd**	Malonyl-CoA decarboxylase
**Ppif**	Peptidyl-prolyl cis-trans isomerase F, mitochondrial
**Nudt19**	Nucleoside diphosphate-linked moiety X motif 19
**Acss1**	Acetyl-coenzyme A synthetase 2-like, mitochondrial
**Acot2**	Acyl-CoA thioesterase 2
**Sfxn5**	Sideroflexin 5
**Atg4d**	Cysteine protease ATG4D Cysteine protease ATG4D, mitochondrial
**Cars2**	Cysteinyl-tRNA synthetase 2 (mitochondrial)(putative)
**Nipsnap1**	Nipsnap homolog 1
**Dock8**	Dedicator of cytokinesis protein 8
**Them4**	Acyl-coenzyme A thioesterase THEM4
**Hpdl**	4-Hydroxyphenylpyruvate dioxygenase-like protein
**Mmaa**	Methylmalonic aciduria type A homolog, mitochondrial
**Pld6**	Mitochondrial cardiolipin hydrolase
**Kcnj11**	ATP-sensitive inward rectifier potassium channel 11
**Lyrm9**	LYR motif-containing protein 9
**Adcy10**	Adenylate cyclase type 10

Proteome analysis (Forner et al, 2009)	
**Gene**	**Description**
**UCP1**	Mitochondrial brown fat uncoupling protein 1
**LETMD1**	Isoform 1 of LETM1 domain-containing protein 1;Isoform 2 of LETM1 domain-containing protein 1;Isoform 3 of LETM1 domain-containing protein 1
**NDUFS7**	NADH dehydrogenase [ubiquinone] iron-sulfur protein 7, mitochondrial precursor
**NDUFS8**	NADH dehydrogenase [ubiquinone] iron-sulfur protein 8, mitochondrial precursor
**APOO**	Apolipoprotein O<br>Novel transmembrane domain containing protein
**UQCR10**	Cytochrome b-c1 complex subunit 9;7 kDa
**COX6B1**	Cytochrome c oxidase subunit VIb isoform 1
**NDUFB11**	NADH dehydrogenase [ubiquinone] 1 beta subcomplex subunit 11, mitochondrial precursor
**ETFDH**	Electron transfer flavoprotein-ubiquinone oxidoreductase, mitochondrial precursor
**SDHA**	Succinate dehydrogenase [ubiquinone] flavoprotein subunit, mitochondrial precursor
**GPD2**	Glycerol-3-phosphate dehydrogenase, mitochondrial precursor; Glycerol phosphate dehydrogenase 2, mitochondrial
**KIAA0564**	hypothetical protein LOC219189;hypothetical protein LOC219189
**NDUFS1**	NADH-ubiquinone oxidoreductase 75 kDa subunit, mitochondrial precursor
**SDHB**	Succinate dehydrogenase [ubiquinone] iron-sulfur subunit, mitochondrial precursor
**MRPL4**	Mitochondrial 39S ribosomal protein L4;20 kDa protein
**COASY**	Bifunctional coenzyme A synthase
**AARS2**	Probable alanyl-tRNA synthetase, mitochondrial precursor
**NDUFB10**	NADH dehydrogenase [ubiquinone] 1 beta subcomplex subunit 10
**NDUFB8**	NADH dehydrogenase [ubiquinone] 1 beta subcomplex subunit 8, mitochondrial precursor
**NDUFV1**	NADH dehydrogenase [ubiquinone] flavoprotein 1, mitochondrial precursor
**NDUFB4**	NADH dehydrogenase [ubiquinone] 1 beta subcomplex subunit 4
**UQCRH**	Cytochrome b-c1 complex subunit 6, mitochondrial precursor
**BRP44**	Brain protein 44
**TSFM**	Elongation factor Ts, mitochondrial precursor
**LACE1**	Lactation elevated protein 1;Protein
**ACSL5**	Long-chain-fatty-acid--CoA ligase 5

Supplementary Figure 2. Genes identified from analysis of cold-inducible mitochondrial transcriptome and proteome in BAT. Genes that are upregulated in both the transcriptome and proteome datasets are shaded in grey.

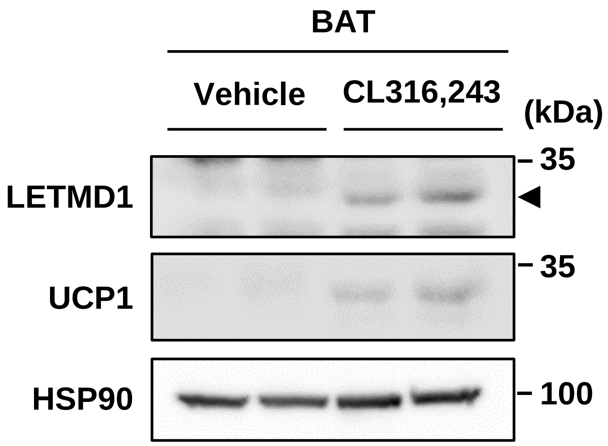

Supplementary Figure 3. LETMD1 protein expression is upregulated by activation of the β3-adrenergic signaling pathway in BAT. Western blot analysis of LETMD1 (arrowhead) and UCP1 protein expression in BAT from adult male mice that were intraperitoneally injected with either vehicle or CL316,243 (1 mg/kg) daily for 5 consecutive days. n=2 per group. Representative images from three independent repeats. The results indicate that LETMD1 protein expression is upregulated by activation of the β3-adrenergic signaling pathway, as evidenced by increased LETMD1 protein expression in BAT from mice treated with CL316,243 compared to vehicle-treated controls. UCP1 protein expression was also increased in response to CL316,243 treatment, consistent with its known role in BAT thermogenesis. HSP90 is loading control. Mice maintained at thermoneutrality (30°C).

Genotype	Number of pups	Percentage(%)
Letmd1 $^{+/+}$	12	24
Letmd1 $^{+/-}$	25	50
Letmd1 $^{-/-}$	13	26
**Total**	**50**	**100**

Supplementary Figure 4. Genotype analysis of pups from *Letmd1* heterozygous KO mice intercrosses shows a Mendelian ratio. *Letmd1* heterozygous KO (*Letmd1* +/-) mice were intercrossed and resulting litters were genotyped at 3 weeks of age. The results show a mendelian ratio of genotypes, indicating the absence of postnatal lethality.

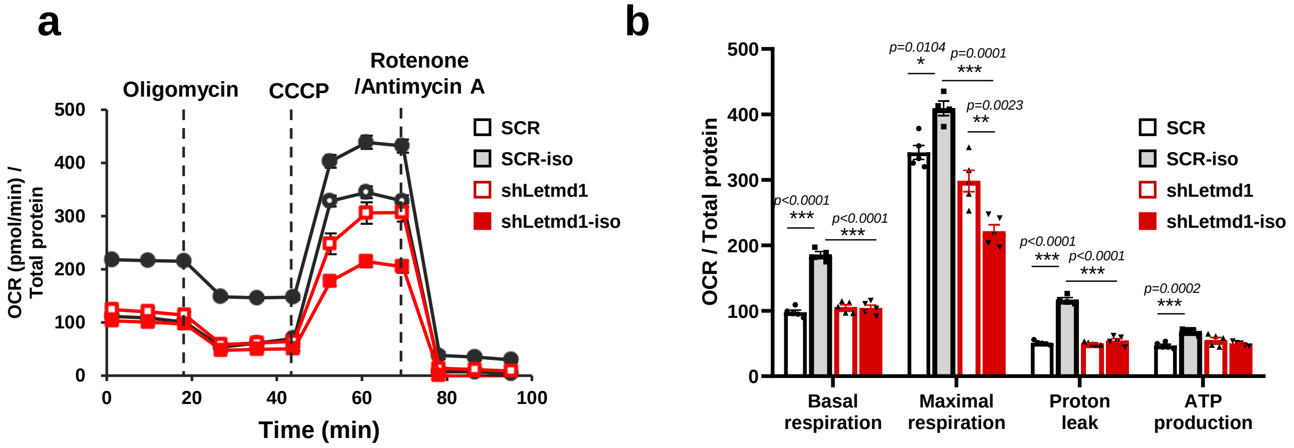

Supplementary Figure 5. LETMD1 deficiency impairs mitochondrial respiration in activated brown adipocytes. a, Oxygen consumption rate (OCR) of control (SCR) and *Letmd1* knockdown (shLetmd1) iBPA cells that were differentiated into brown adipocytes. Cells were treated with 1 µM isoproterenol (iso) for 1 h before performing extracellular flux analysis (XFe96, Seahorse Bioscience). OCR values were normalized to the total protein levels of each well. $n_{SCR}=5$, $n_{SCR\text{-}iso}=4$, $n_{shLetmd1}=5$, $n_{shLetmd1\text{-}iso}=5$. **b,** Basal respiration, maximal respiration, proton leak, and ATP production in same sets as **a**. Data presented as mean ± SEM. $*p < 0.05$, $** p < 0.005$, $*** p < 0.0005$. The significance of the results was assessed using one-way ANOVA. Source data are provided as a Source Data file.

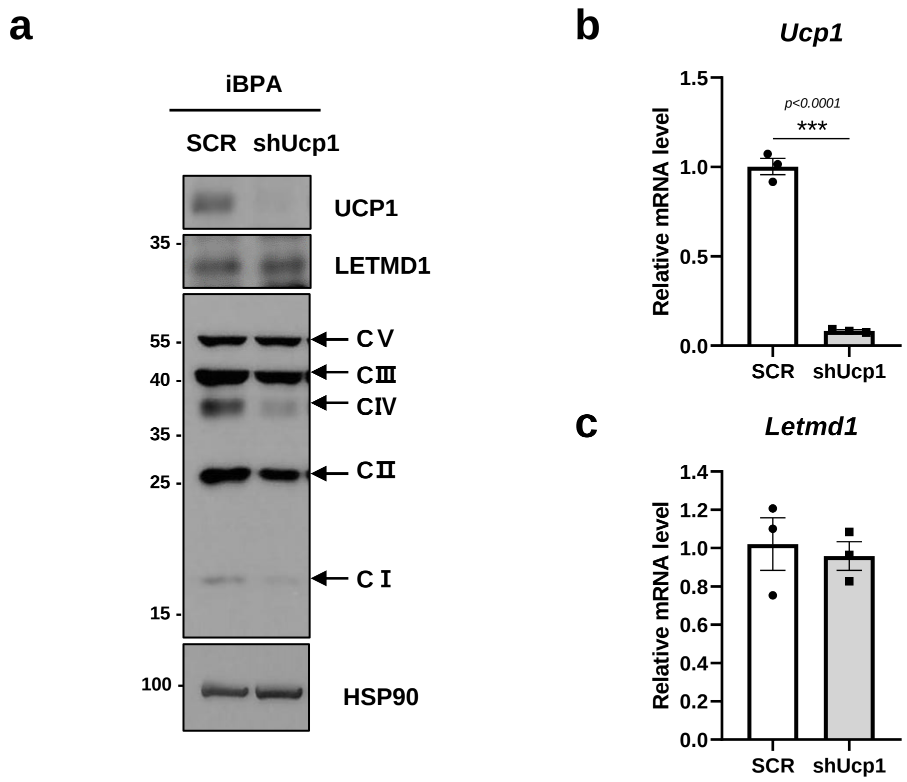

Supplementary Figure 6. Loss-of-UCP1 does not affect LETMD1 expression. a, Western blot analysis of UCP1, LETMD1 and OXPHOS complex proteins in control (SCR) and *Ucp1* knockdown (shUcp1) brown adipocyte lysates. Representative images from three independent repeats. HSP90 is a loading control. **b and c,** mRNA expression of *Ucp1* (b) and *Letmd1* (c) in total RNA from SCR and shUcp1 brown adipocytes. n=3 per group. Data presented as mean ± SEM. *** $p < 0.0005$. The significance of the results was assessed using a two-tailed Student' *t*-test. Source data are provided as a Source Data file.

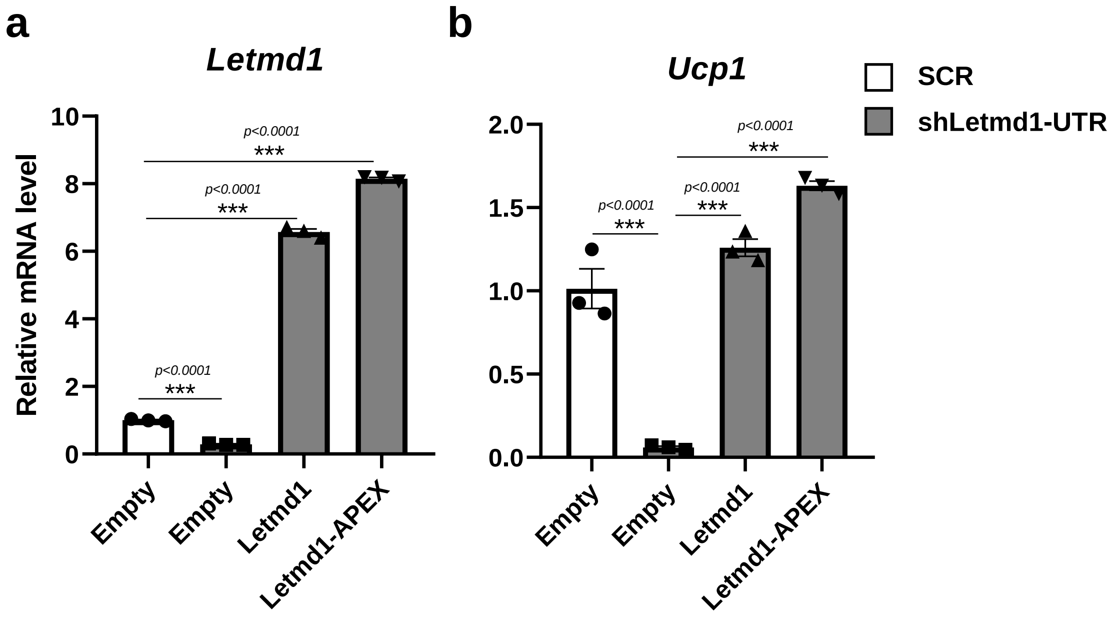

Supplementary Figure 7. Letmd1-APEX2 fusion protein expression restores *Ucp1* expression in Letmd1 knockdown iBPA brown adipocytes. a and b, mRNA expression of *Letmd1* (a) and *Ucp1* (b) in differentiated iBPA brown adipocytes from indicated conditions. n=3 per group. The results show that the expression of either Letmd1-APEX2 fusion or Letmd1 can effectively restore *Ucp1* expression in shLetmd1-UTR knockdown iBPA brown adipocytes. To generate shLetmd1 knockdown iBPA cells, a lentiviral vector (pLVX-EGFP-U6, VectorBuilder) containing an shRNA sequence targeting the 3'-UTR (shLetmd1-UTR) of *Letmd1* mRNA or a scrambled (SCR) shRNA sequence was stably transduced to iBPA cells. These cells were then transduced with lentiviruses produced from pLVX-IRES-ZsGreen1 (Empty), pLVX-IRES-ZsGreen1-Letmd1 (Letmd1), and pLVX-IRES-ZsGreen1-Letmd1-APEX2 (Letmd1-APEX2) and differentiated into brown adipocytes. The sequence used to create shLetmd1-UTR knockdown iBPA cells is provided: 5'-GCATCTCTGGTACTGAACTTTCT CGAGAAAGTTCAGTACCAGA GATGC - 3'. Data presented as mean ± SEM. *** $p < 0.0005$. . The significance of the results was assessed using one-way ANOVA. Source data are provided as a Source Data file.

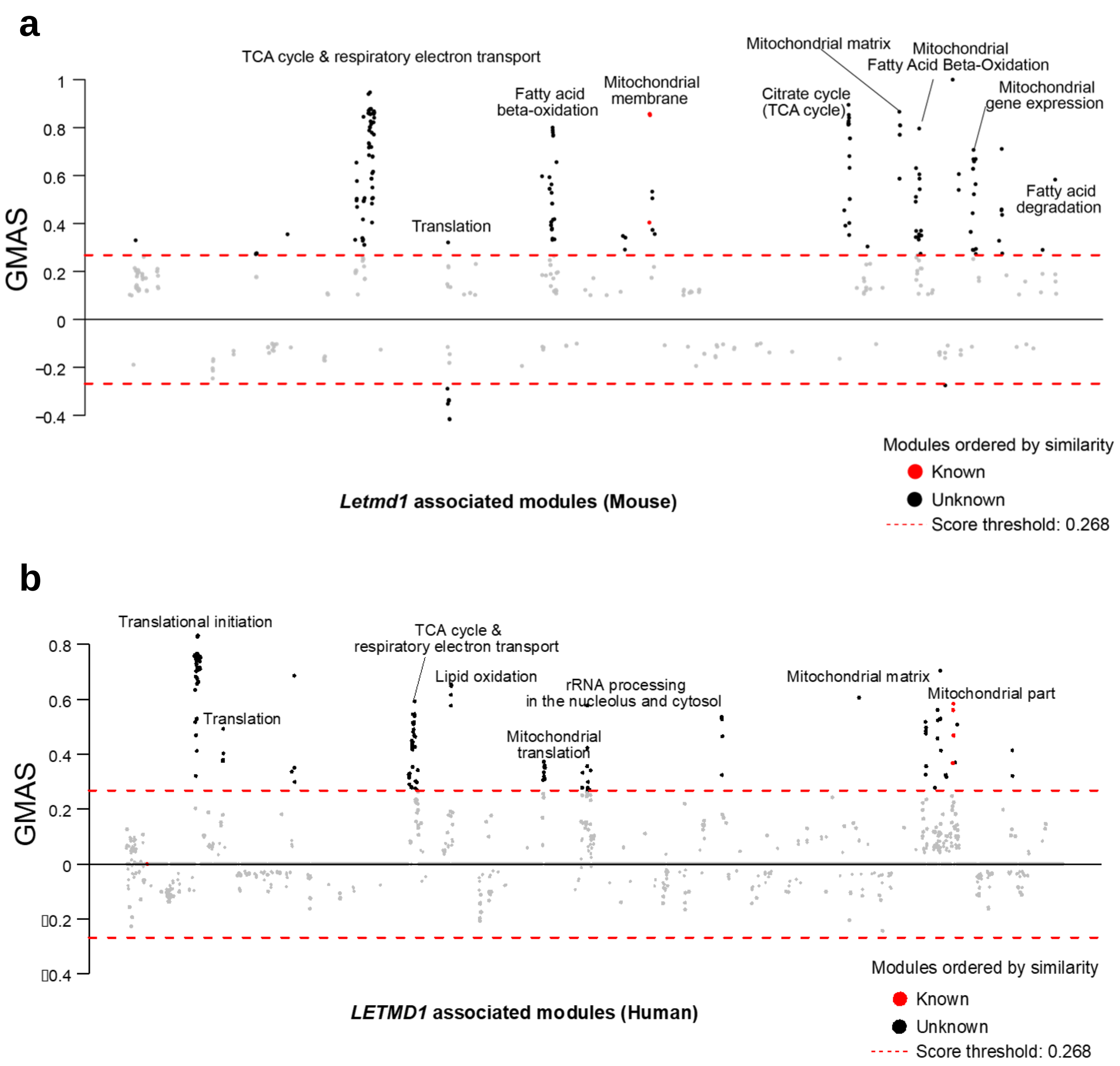

Supplementary Figure 8. *Letmd1*-associated GO terms or gene modules in adipose tissues. a, Gene modules in mouse adipose tissues. **b**, Gene modules in human adipose tissues. The enriched gene modules are calculated by the Gene-Module Association Determination (G-MAD) approach according to the inter-gene correlations. The threshold is represented by the red dashed line (absolute Gene-Module Association Score (GMAS) >= 0.268). Terms above the threshold are identified as the significant associated terms of *Letmd1* expression in adipose tissues and GO terms or gene modules are ranked by similarity. Known *Letmd1*-associated terms are shown as red dots and new significant associated terms are colored in black (data from https://systems-genetics.org/gmad (PMID: 31754022)).

Gene	Forward	Reverse
Letmd1	5'-CAGATGTTATGGGCTGATGG-3'	5'-CTCGTCGGAACTGTCTCAAA-3'
Ucp1	5'-CTTTGCCTCACTCAGGATTGG-3'	5'-AATGACTGGAGGTGTGGCAGT-3'
Pgc1α	5'-CAAAGCTGGCTTCAGTCACA-3'	5'-AAAAGTAGGCTGGGCTGTCA-3'
Prdm16	5'-CAGCACGGTGAAGCCATTC-3'	5'-GCGTGCATCCGCTTGTG-3'
Dio2	5'-CAACTGCCAGCCTTCCGCCA-3'	5'-CAGGCACTGCCCAGCCATCC-3'
Cidea	5'-CTCGGCTGTCTCAATGTCAA-3'	5'-GGAACTGTCCCGTCATCTGT-3'
Cox8b	5'-GCGAAGTTCACAGTGGTTCC-3'	5'-GGAACCATGAAGCCAACGAC-3'
Rpl32	5'-GGCCTCTGGTGAAGCCCAAGATCG-3'	5'-CCTCTGGGTTTCCGCCAGTTTCGC-3'

Supplementary Table 1. Primers for qRT-PCR used in this study.

Supplementary Figure 3 (uncropped scans)

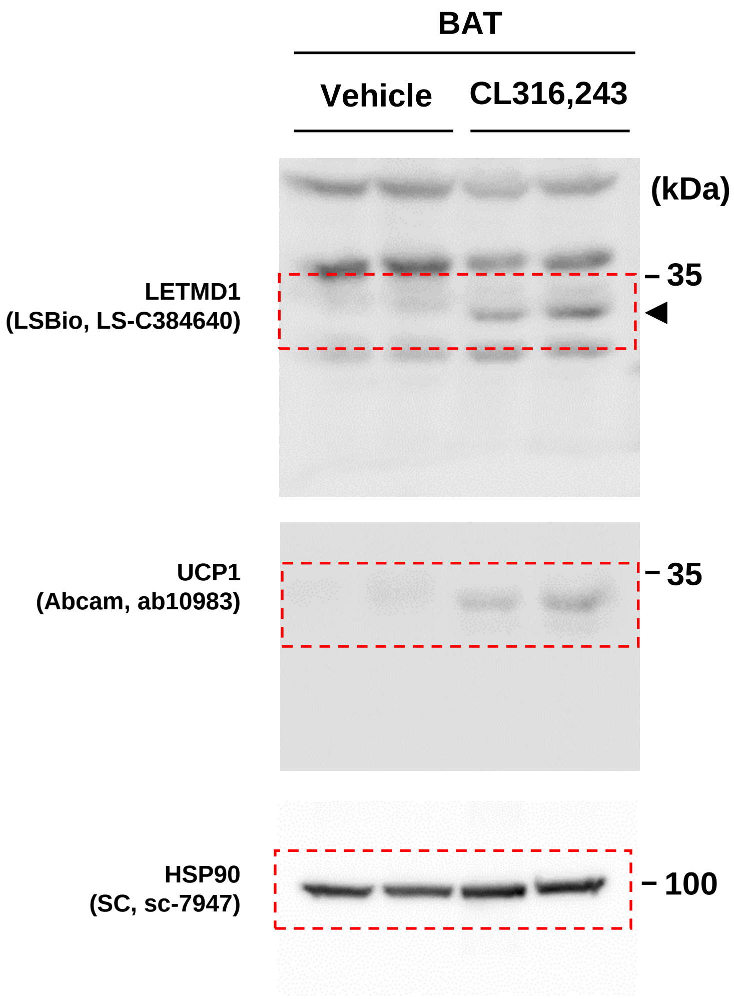

Supplementary Figure 6 (uncropped scans)

a

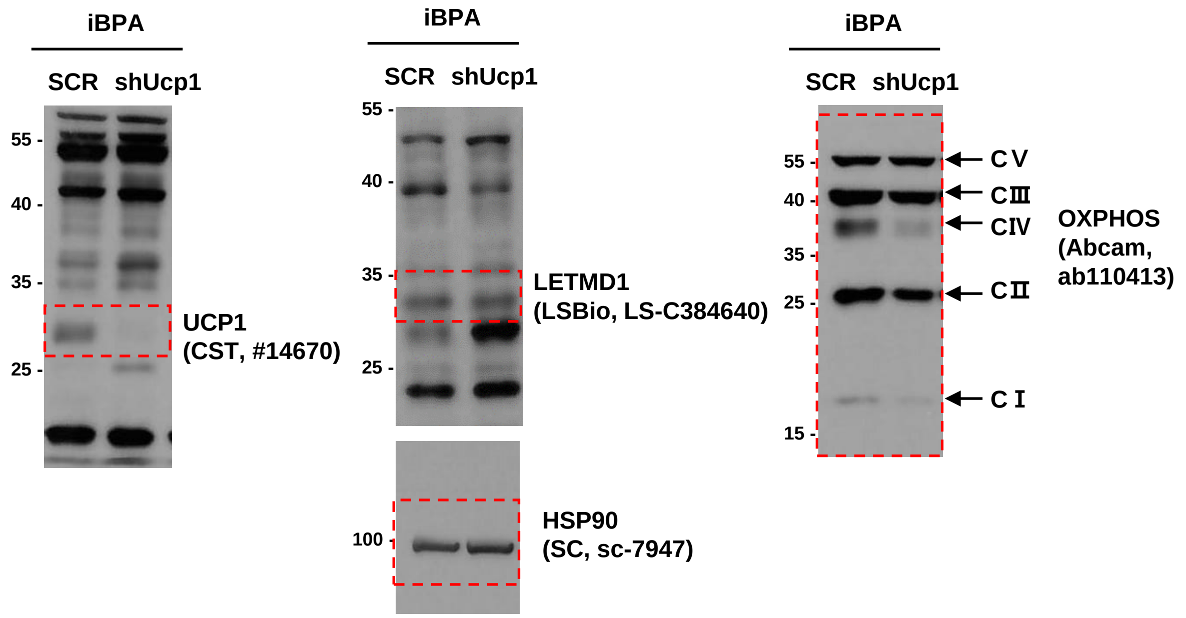

Supplement: Supplementary file 1 — Supplementary Information [file 41467_2023_39106_MOESM1_ESM.pdf]
