# Supplementary material for: Mitochondrial matrix protein LETMD1 maintains thermogenic capacity of brown adipose tissue in male mice
Source: Nat Commun. 2023 Jun 23;14:3746. doi: 10.1038/s41467-023-39106-z (PMC10290150; doi:10.1038/s41467-023-39106-z)
Supplement: Supplementary file 2 — Reporting Summary [file 41467_2023_39106_MOESM2_ESM.pdf]

nature portfolio

Corresponding author(s): Jae Myoung Suh, Kwang-Hee Bae, Won Kon Kim

Last updated by author(s): May 4, 2023

Reporting Summary

Nature Portfolio wishes to improve the reproducibility of the work that we publish. This form provides structure for consistency and transparency in reporting. For further information on Nature Portfolio policies, see our Editorial Policies and the Editorial Policy Checklist.

Statistics

For all statistical analyses, confirm that the following items are present in the figure legend, table legend, main text, or Methods section.

n/a	Confirmed	
☐	☒	The exact sample size (*n*) for each experimental group/condition, given as a discrete number and unit of measurement
☐	☒	A statement on whether measurements were taken from distinct samples or whether the same sample was measured repeatedly
☐	☒	The statistical test(s) used AND whether they are one- or two-sided<br>*Only common tests should be described solely by name; describe more complex techniques in the Methods section.*
☐	☒	A description of all covariates tested
☐	☒	A description of any assumptions or corrections, such as tests of normality and adjustment for multiple comparisons
☐	☒	A full description of the statistical parameters including central tendency (e.g. means) or other basic estimates (e.g. regression coefficient) AND variation (e.g. standard deviation) or associated estimates of uncertainty (e.g. confidence intervals)
☐	☒	For null hypothesis testing, the test statistic (e.g. F, t, r) with confidence intervals, effect sizes, degrees of freedom and P value noted<br>*Give P values as exact values whenever suitable.*
☒	☐	For Bayesian analysis, information on the choice of priors and Markov chain Monte Carlo settings
☐	☒	For hierarchical and complex designs, identification of the appropriate level for tests and full reporting of outcomes
☐	☒	Estimates of effect sizes (e.g. Cohen's *d*, Pearson's *r*), indicating how they were calculated

Our web collection on statistics for biologists contains articles on many of the points above.

Software and code

Policy information about availability of computer code

Data collection	The following software were used for data collection:<br>CFX Maestro Software 2.0 (Bio-Rad)<br>Wave 2.6.1 Software (Agilent)
Data analysis	The following software were used for data analysis:<br>CFX Maestro Software 2.0 (Bio-Rad), Wave 2.6.1 Software (Agilent)<br>Prism 8.4 Software (GraphPad), AnalyzIR software (FOTRIC)

For manuscripts utilizing custom algorithms or software that are central to the research but not yet described in published literature, software must be made available to editors and reviewers. We strongly encourage code deposition in a community repository (e.g. GitHub). See the Nature Portfolio guidelines for submitting code & software for further information.

Data

Policy information about availability of data

All manuscripts must include a data availability statement. This statement should provide the following information, where applicable:

- Accession codes, unique identifiers, or web links for publicly available datasets
- A description of any restrictions on data availability
- For clinical datasets or third party data, please ensure that the statement adheres to our policy

We analyzed publicly available gene expression data for genes that are enriched in BAT, as compared to epididymal WAT (eWAT) (GSE92844) and induced by cold stimulus (GSE70437). RNA-sequencing data are deposited in Gene Expression Omnibus (GEO) under accession number GSE. The source data for this study are provided with this paper. Additional data that support the findings of this study are available from the corresponding author upon reasonable request.

Human research participants

Policy information about studies involving human research participants and Sex and Gender in Research.

Reporting on sex and gender	N/A
Population characteristics	N/A
Recruitment	N/A
Ethics oversight	N/A

Note that full information on the approval of the study protocol must also be provided in the manuscript.

Field-specific reporting

Please select the one below that is the best fit for your research. If you are not sure, read the appropriate sections before making your selection.

☒ Life sciences ☐ Behavioural & social sciences ☐ Ecological, evolutionary & environmental sciences

For a reference copy of the document with all sections, see nature.com/documents/nr-reporting-summary-flat.pdf

Life sciences study design

All studies must disclose on these points even when the disclosure is negative.

Sample size	For our in vivo experiments, 3-6 mice were sacrificed per experiment group. Specific sample size for each experiment is indicated in the manuscript. This sample size was determined based on the minimum number of animals being sacrificed while at the same time, fulfilling statistically.<br>Statistical analysis was performed using GraphPad Prism 8.4 software (GraphPad). All statistics are described in figure legends. In general, comparisons between two groups were performed using the two-tailed Student's t test, and multiple group comparisons were performed by one-way ANOVA followed by Tukey-Kramer post-hoc test.
Data exclusions	For our experiment, we used ethically minimum number of mice that are statistically significant. Each group of mice were housed in controlled environment and they were of same gender and age. Therefore, we did not exclude any mouse from any group in the experiments that were carried out.
Replication	All experiments conducted in this study were reproducible through repeated experiments. We added the number of independent replicates performed in the experiments to the legends.
Randomization	All mice and cells were allocated in random. No bias in sample allocation was involved.
Blinding	Investigators were blinded to group allocation during data collection and analysis

Reporting for specific materials, systems and methods

We require information from authors about some types of materials, experimental systems and methods used in many studies. Here, indicate whether each material, system or method listed is relevant to your study. If you are not sure if a list item applies to your research, read the appropriate section before selecting a response.

Materials & experimental systems

n/a	Involved in the study
☐	☒ Antibodies
☐	☒ Eukaryotic cell lines
☒	☐ Palaeontology and archaeology
☐	☒ Animals and other organisms
☒	☐ Clinical data
☒	☐ Dual use research of concern

Methods

n/a	Involved in the study
☒	☐ ChIP-seq
☒	☐ Flow cytometry
☒	☐ MRI-based neuroimaging

Antibodies

Antibodies used

Antibodies used in immunoblot analyses included those against LETMD1 (LSBio, LS-C384640, 1:1000); UCP1 (Abcam, ab10983, 1:1000); HSP90 (Santa Cruz, sc-7947, 1:2000); α-Tubulin (Sigma-Aldrich, sc-8035, 1:3000); OXPHOS cocktail (Abcam, ab110413, 1:1000); Streptavidin-HRP (Thermo Scientific, S911, 1:3000); and Flag (Sigma-Aldrich, F3165, 1:3000). The specific signals were amplified by horseradish peroxidase-conjugated secondary anti-rabbit or anti-mouse antibody (Santa Cruz, 1:3000).

Validation

All the antibodies were validated for the species and imunoblotting by the correspondent manufacturer, which is described in the manufacturer's website.

- LETMD1 anitbody used was validated for immuno-blotting, immunohistochemistry and immunofluorescence.
(https://www.lsbio.com/antibodies/letmd1-antibody-hccr1-antibody-if-immunofluorescence-ihc-wb-western-ls-c335200/345559)
- UCP1 anitbody used was validated for immuno-blotting and immunohistochemistry.
(https://www.abcam.com/UCP1-antibody-ab10983.html?gclsrc=aw.ds|
aw.ds&gclid=Cj0KCQjw39uYBhCLARIsAD_SzMR_2Su6DRO4AuBAWmmmlAKEbPf6yYBgeVq0qF9YIr--KeznWyrvXOcaAvTqEALw_wcB)
- HSP90 anitbody used was validated for WB, IP, IF, IHC(P), FCM and ELISA.
(https://www.scbt.com/ko/p/hsp-90alpha-beta-antibody-f-8)
- α-Tubulin anitbody used was validated for WB, IF, and IHC.
(https://www.sigmaaldrich.com/KR/ko/product/sigma/t5168)
- Anti-OXPHOS cocktail used was validated for WB.
(https://www.abcam.com/total-oxphos-rodent-wb-antibody-cocktail-ab110413.html)
- HRP-Conjugated Streptavidin consists of streptavidin protein that is covalently conjugated to horseradish peroxidase (HRP) enzyme (RZ > 3.0). Streptavidin binds to biotin and the conjugated HRP provides enzyme activity for detection using an appropriate substrate system. This particular product has been used primarily in sandwich ELISA applications to provide consistent measurement of biotinylated detection antibodies.
(https://www.thermofisher.com/order/catalog/product/N100)
- Monoclonal ANTI-FLAG® M2 antibody produced in mouse has been used in: immunoblotting, immunoprecipitation, immunocytochemistry, immunofluorescence, ELISA, EIA, chromatin immunoprecipitation, electron microscopy, flow cytometry
(https://www.sigmaaldrich.com/KR/ko/product/sigma/f3165)
supershift assays

Eukaryotic cell lines

Policy information about cell lines and Sex and Gender in Research

Cell line source(s)

We have provided the source of all the cell lines used in the Methods section.
An immortalized brown preadipocyte (iBPA) cell line was provided by Dr. Shingo Kajimura (UCSF, San Francisco, CA, USA) .
GP2-293 Packaging Cell Line (631458, Clontech)
HEK293T (CRL-3216, ATCC)

Authentication

Cells were authentificated based on their morphology, growth condition and specific gene expression.

Mycoplasma contamination

All cell lines used in this study were tested negative for mycoplasma contamination.

Commonly misidentified lines
(See ICLAC register)

No commonly misidentified cell lines were used in the study.

Animals and other research organisms

Policy information about studies involving animals; ARRIVE guidelines recommended for reporting animal research, and Sex and Gender in Research

Laboratory animals

Specific pathogen–free C57BL/6N mice were purchased from KOATECH (South Korea). Specific pathogen–free CTB-Flpe mice (JAX 003800), Ucp1-Cre (JAX 024670) mice were purchased from Jackson Laboratory (Jackson Labs, Bar Harbor, ME).
In our study, only male mice were used and their specific age is mentioned in the manuscript.
Mice were housed under 12 light/12 dark cycle, temperatures of 22±2°C with 50±10% humidity.

The age of the mice used in the experiments is as follows:
Figure 1. (c) C57BL/6N, male, 8-week-old

Figure 1. (d,e) C57BL/6N, male, postnatal day1, day7, day14
Figure 1. (f,g) C57BL/6N, male, 4-month-old
Figure 1. (h,j) C57BL/6N, male, 11-week-old
Figure 3. (a-c) WT and Letmd1 KO, male, 13-week-old
Figure 3. (d-f) WT and Letmd1 KO, male, 13-week-old
Figure 3. (g) WT and Letmd1 KO, male, postnatal day1, day2, day3
Figure 3. (h,i) WT and Letmd1 KO, male, 14-week-old
Figure 3. (j,k) WT and Letmd1 KO, male, 15-week-old
Figure 3. (i) WT and Letmd1 KO, male, 10-week-old
Figure 4. (a) WT and Letmd1 KO, male, 5-week-old
Figure 4. (b) WT and Letmd1 KO, male, 5-month-old
Figure 4. (c) WT and Letmd1 KO, embryonic day16,5
Figure 4. (d) WT and Letmd1 KO, male, postnatal day1, day2, day3
Figure 4. (e) WT and Letmd1 KO, male, 7-week-old
Figure 5. (a,b) WT and Letmd1 KO, male, 5-month-old
Figure 5. (c-f) WT and Letmd1 KO, male, 13-week-old
Figure 6. (b-k) WT and Letmd1 BKO, male, 12-week-old
Figure S3. C57BL/6N, male, 14-week-old
Figure S4. WT and Letmd1 KO, male and female, 3-week-old

Wild animals

No wild animals were captured for our study. Our mice samples were provided by approved mouse vendor.

Reporting on sex

We used only male mice for following animal intervention experiments for the concern of the estrogen cycle fluctuation might affect the experimental results.

Field-collected samples

No wild animals were field-collected in our study.

Ethics oversight

All mouse experiments were approved and performed under institutional guidelines of the Korean Research Institute of Biotechnology and Bioscience.

Note that full information on the approval of the study protocol must also be provided in the manuscript.
